# Regulated cell death in ovarian cancer: molecular mechanisms and therapeutic targets

**DOI:** 10.3389/fcell.2026.1910706

**Published:** 2026-08-07

**Authors:** Yu Liu, Zhixing Geng, Baofu Jia, Xinxin Wang, Hongge Ju, Junxia Bao, Xiaoqin Liao, Xiaopeng Deng, Ye Guo, Fu Lin

**Affiliations:** 1 School of Pharmacy, Baotou Medical College, Baotou, China; 2 School of Basic Medicine and Forensic Science, Baotou Medical College, Baotou, China

**Keywords:** cancer therapy, mechanisms, ovarian cancer, regulated cell death, small-molecule compounds

## Abstract

Ovarian cancer (OC) poses a significant threat to women’s health due to its high malignancy and poor prognosis, with limited efficacy of existing targeted therapies. In recent years, the elucidation of molecular mechanisms underlying regulated cell death (RCD) has substantially broadened our understanding of OC pathogenesis and therapeutic resistance, rendering the modulation of RCD pathways by small-molecule compounds a highly promising therapeutic strategy. This review systematically outlines the mechanistic underpinnings of multiple major RCD subtypes in OC, with a focused summary of advances in innovative small-molecule compounds targeting these pathways; discusses nanodelivery platforms for RCD modulation, predictive biomarkers for patient stratification, and the current landscape of clinical trials targeting RCD in OC; and finally analyzes the opportunities and challenges in clinical translation within this field, aiming to provide novel insights and directions for precision therapy in OC.

## 1 Introduction

Ovarian cancer (OC) constitutes the eighth most prevalent malignancy in women globally and remains among the principal contributors to gynecologic cancer mortality (Caruso et al., 2025). Approximately 90% of ovarian malignancies are of epithelial origin, with high-grade serous OC (HGSC) representing the predominant histologic subtype, accounting for 70%–80% of cases. Owing to the insidious onset of early symptoms and the absence of effective screening modalities, the majority of patients are diagnosed at advanced stages, resulting in a 5-year survival rate of less than 30% (Alizadeh et al., 2025). Recent advances in elucidating the molecular heterogeneity of OC and its tumor microenvironment-mediated regulatory mechanisms have catalyzed the clinical integration of novel therapeutic strategies beyond conventional platinum-based chemotherapy, exemplified by poly-ADP-ribose polymerase inhibitors (PARPi), immune checkpoint inhibitors, and additional targeted agents (Xu et al., 2026). Nevertheless, these modalities are frequently encumbered by systemic toxicities, intrinsic or acquired resistance, and high recurrence rates, substantially constraining their long-term efficacy (Xu et al., 2026). Consequently, the imperative to develop next-generation interventions grounded in deeper mechanistic insights and superior therapeutic efficacy has become increasingly urgent.

Regulated cell death (RCD), also referred to as programmed cell death, constitutes a genetically encoded, biochemically orchestrated form of cell death that is fundamentally distinct from stochastic accidental cell death (Peng et al., 2022). RCD serves as a central regulatory mechanism in embryonic development, tissue homeostasis maintenance, immune surveillance, and the pathophysiological progression of diverse diseases, particularly malignancies (Kayagaki et al., 2024). With deepening investigations, the conceptual scope of RCD has undergone continuous expansion: from classical apoptosis, autophagy, necroptosis and pyroptosis, to metal ion-dependent modalities exemplified by ferroptosis and cuproptosis, and further to emerging forms including parthanatos and disulfidptosis (Meng et al., 2025). These cumulative discoveries have not only illuminated the intricate regulatory networks through which cells respond to diverse stress signals, but also furnished novel perspectives for elucidating tumorigenesis. Accumulating evidence indicates that distinct RCD subtypes constitute critical hallmarks of OC initiation and progression, ultimately furnishing a rationale for establishing differential therapeutic strategies (Peng et al., 2022; Ruan et al., 2023). To date, diverse small-molecule compounds targeting specific RCD subtypes have emerged as a promising therapeutic avenue, with rapid advances being achieved in OC.

Despite extensive research on RCD across multiple malignancies (Chen et al., 2021; Zhang et al., 2023), a systematic synthesis of RCD pathways in OC remains elusive. This review summarizes the molecular mechanisms of diverse RCD subtypes implicated in OC, with an emphasis on small-molecule compounds targeting these pathways; discusses nanodelivery platforms for RCD modulation, predictive biomarkers for patient stratification, and the current landscape of clinical trials targeting RCD in OC; and finally addresses the challenges and future directions in this field. We aim to provide a systematic framework for understanding RCD biology in OC and to offer insights facilitating its therapeutic translation.

## 2 Classical cell death

### 2.1 Apoptosis

**FIGURE 1:**
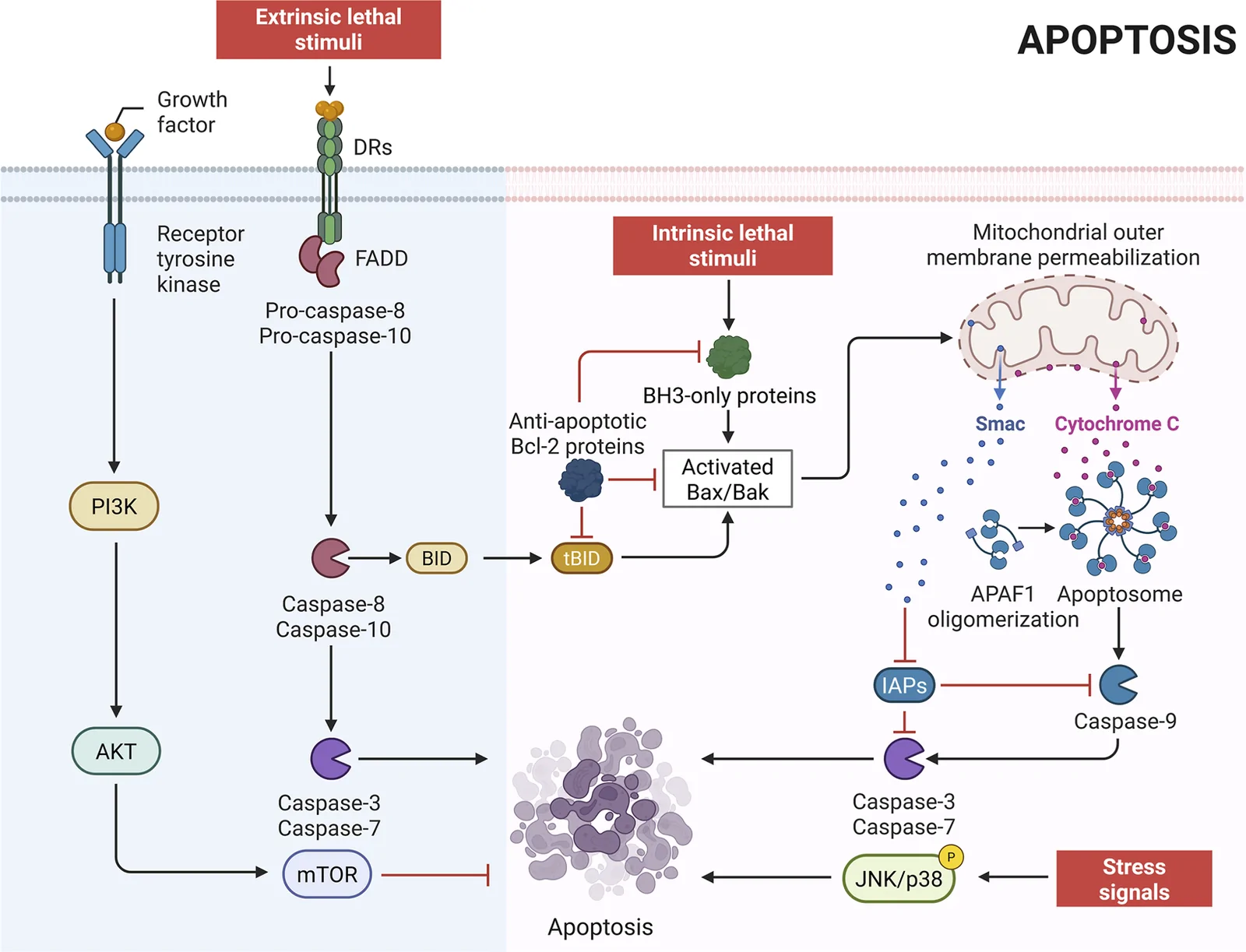
Core apoptotic pathways in OC, comprising intrinsic and extrinsic pathways. Abbreviations: FADD, Fas-associated death domain; BID, BH3-interacting domain death agonist; tBID, truncated BID; APAF1, apoptotic protease activating factor 1.

Apoptosis represents the classical form of RCD, characterized by distinctive morphological features including cell shrinkage, chromatin condensation, nuclear fragmentation, and ultimately the formation of membrane-enclosed apoptotic bodies (Figure 1) (Smith and Hergenrother, 2024). This process is governed by precise molecular signaling pathways, broadly categorized into extrinsic and intrinsic pathways. The extrinsic pathway is initiated by the engagement of tumor necrosis factor (TNF) family ligands with cell surface death receptors (DRs). Upon receptor activation, the adaptor protein Fas-associated protein with death domain is recruited, leading to caspase-8 activation and subsequent cleavage of the effector caspase-3, thereby executing cell death (Smith and Hergenrother, 2024). The intrinsic pathway, conversely, is triggered by mitochondrial stress signals, wherein pro-apoptotic proteins Bax (Bcl-2-associated X protein) and Bak (Bcl-2-homologous antagonist/killer) drive mitochondrial outer membrane permeabilization, facilitating cytochrome c release into the cytosol. Cytochrome c subsequently associates with Apaf-1 to form the apoptosome, activating caspase-9, which through a proteolytic cascade likewise activates caspase-3 to ultimately execute the apoptotic program (Lin F. et al., 2024).

#### 2.1.1 Targeting Bcl-2 family proteins

The B-cell lymphoma 2 (Bcl-2) family proteins serve as the central regulatory hub of the intrinsic pathway, governing cell fate through modulation of mitochondrial outer membrane permeability. Based on structural and functional divergence, family members are categorized into three distinct classes: anti-apoptotic proteins, pro-apoptotic effectors, and pro-apoptotic BH3-only proteins (Birkinshaw and Czabotar, 2017). Under physiological conditions, anti-apoptotic proteins maintain cellular survival by sequestering and inhibiting pro-apoptotic effectors and BH3-only proteins. Upon apoptotic stimulation, however, activated BH3-only proteins either directly activate pro-apoptotic effectors or neutralize anti-apoptotic proteins, thereby promoting Bax/Bak oligomerization and pore formation on the mitochondrial outer membrane, ultimately culminating in cytochrome c release and apoptotic execution (Czabotar et al., 2014). Aberrant expression of Bcl-2 family proteins is intimately linked to therapeutic resistance during OC progression (Marone et al., 1998). Consequently, selective targeting of these proteins effectively restores apoptotic sensitivity in cancer cells, holding considerable promise for benefiting patients with OC.

##### 2.1.1.1 BH3 mimetics

BH3 mimetics constitute a class of small-molecule compounds that functionally mimic BH3-only proteins by selectively engaging and neutralizing anti-apoptotic proteins, thereby liberating pro-apoptotic effectors to induce mitochondrial outer membrane permeabilization and activate the caspase cascade, ultimately culminating in cellular apoptosis (Diepstraten et al., 2022). Currently, multiple BH3 mimetics have entered clinical development (Diepstraten et al., 2022), among which ABT-199 (venetoclax) has gained Food and Drug Administration (FDA) approval for specific hematologic malignancies including chronic lymphocytic leukemia and acute myeloid leukemia, with expanding indications anticipated. In OC therapy, BH3 mimetics have demonstrated considerable promise. For instance, IS21 sensitizes cells to the PARPi olaparib (Valentini et al., 2023); S1, a selective Bcl-2 inhibitor, disrupts glucose metabolism in cells and induces apoptosis through sirtuin 3 activation (Xiang et al., 2016); and ABT-737 induces apoptosis in HGSC explants via B-cell lymphoma-extra large inhibition, with its efficacy dependent on Bim expression. Notably, low expression levels of myeloid cell leukemia-1 (Mcl-1) or phosphorylated extracellular signal-regulated kinase (ERK) enhance drug responsiveness in Bim-positive tumors, and these three factors collectively constitute a critical biomarker signature for predicting BH3 mimetic efficacy (Lheureux et al., 2015). However, highly expressed anti-apoptotic proteins such as Mcl-1 in tumor cells can suppress apoptotic signaling through compensatory mechanisms, thereby rendering mono-targeted BH3 mimetics ineffective (Lheureux et al., 2015). Consequently, the development of “pan-BH3 mimetics” capable of simultaneously engaging multiple anti-apoptotic proteins has emerged as a critical research direction for overcoming therapeutic resistance.

##### 2.1.1.2 Natural compounds

In recent years, natural compounds and their derivatives have garnered significant research interest in modulating OC cell apoptosis, owing to their advantageous profiles including reduced resistance liability and facile preparation. For instance, Zhao et al. demonstrated that the emodin derivative Emd-D directly targets the glycolytic enzyme phosphofructo-2-kinase/fructose-2,6-biphosphatase 4, disrupts glucose metabolism in cells, and promotes apoptosis through the steroid receptor cofactor 3/mechanistic target of rapamycin complex 1 (mTORC1) axis. *In vivo* experiments further revealed that Emd-D treatment suppresses xenograft tumor growth, attenuates tumor glycolysis, and upregulates Bax/Bcl-2 expression (Zhao X. et al., 2025). In a separate study, Zarei et al. employed dendritic curcumin combined with oxaliplatin to treat cells, markedly inducing apoptosis through modulation of Bax and Bcl-2 expression equilibrium (Zarei et al., 2025). Additionally, Su et al. identified that the marine alkaloid isoaaptamine disrupts mitochondrial membrane potential in cells, downregulates Bcl-2 while upregulating Bax, and activates the caspase-9/caspase-3/PARP cascade, thereby triggering apoptosis. This compound concurrently inhibits epithelial-mesenchymal transition (EMT) and blocks key pathways implicated in OC progression and chemoresistance, including phosphoinositide 3-kinase (PI3K)/Akt, mitogen-activated protein kinase (MAPK), and signal transducer and activator of transcription 3 (STAT3) (Su I. L. et al., 2025). Collectively, these findings indicate that natural compounds can not only directly initiate apoptotic programs but also operate through multiple mechanisms encompassing metabolic reprogramming interference, EMT phenotype reversal, and suppression of cell survival pathways, offering novel therapeutic insights for overcoming chemoresistance in OC.

In OC, aberrant expression of Bcl-2 family proteins enables malignant cells to evade apoptosis, exacerbates malignant progression, and diminishes therapeutic sensitivity, culminating in unfavorable patient outcomes (Marone et al., 1998). Small-molecule inhibitors targeting these proteins, despite their considerable potential, frequently encounter clinical translation challenges including off-target toxicity, suboptimal stability, and rapid metabolic clearance (Zhang et al., 2023). Exosomes, as naturally occurring nanovesicular delivery systems, offer a promising strategy to enhance drug stability and mitigate adverse effects, leveraging their inherent biocompatibility and targeting capabilities to improve both the efficacy and safety profiles of Bcl-2 inhibitors (Muttiah et al., 2024). Future optimization of exosome-based carrier technologies, integrated with personalized combination regimens, holds substantial promise for driving significant advances in OC targeted therapy and improving patient survival outcomes.

#### 2.1.2 Targeting IAPs

Inhibitor of apoptosis proteins (IAPs) are frequently overexpressed in various malignancies including OC, an aberration intimately associated with tumor progression, therapeutic resistance, and unfavorable prognosis. Consequently, targeting IAPs has emerged as a promising therapeutic strategy (Plati et al., 2011). As a prominent class of IAP-targeted agents, the second mitochondrial-derived activator of caspases (Smac) mimetics bind to the baculoviral IAP repeat domains of IAPs, thereby alleviating IAP-mediated caspase suppression and promoting tumor cell apoptosis (Zhu et al., 2019). Based on structural distinctions, Smac mimetics are categorized into monomeric and dimeric classes with divergent mechanistic emphases (Wu et al., 2007): monomeric Smac mimetics primarily target X-linked inhibitor of apoptosis (XIAP) to directly restore caspase activity and induce apoptosis, whereas dimeric Smac mimetics, leveraging dual binding sites, more efficiently induce cellular inhibitor of apoptosis proteins 1 and 2 ubiquitination and proteasomal degradation, consequently destabilizing the tumor necrosis factor receptor (TNFR) complex II and activating TNFα-mediated apoptotic signaling—a critical rate-limiting step for apoptosis induction in certain resistant cell populations. In the context of OC therapy, the monomeric Smac mimetic LCL161 has been shown to sensitize BRCA1-mutant cell lines to treatment, an effect attributable to specific XIAP upregulation via the nuclear factor kappaB (NF-κB) pathway (Cremona et al., 2022). In a separate study, the monomeric Smac mimetic AT406 targets IAPs as the terminal effector of the collagen type XI alpha 1 (COL11A1)-activated integrin α1β1/discoidin domain receptor 2-Src-PI3K/Akt-NF-κB signaling axis, effectively reversing cisplatin resistance in preclinical models and offering a novel strategy for COL11A1-overexpressing resistant OC (Rada et al., 2018). Furthermore, the dimeric Smac mimetic birinapant, in combination with carboplatin, synergistically induces apoptosis in a subset of platinum-resistant epithelial OC cells. The underlying mechanism involves dual actions: birinapant not only directly antagonizes IAPs to alleviate caspase suppression but also triggers TNFα autocrine/paracrine signaling. High-throughput screening utilizing 3D organoids revealed that this regimen exhibits efficacy in approximately half of platinum-resistant cell lines and patient-derived primary samples, with therapeutic activity further validated in a platinum-resistant patient-derived xenograft model (Singh et al., 2022). Collectively, these findings demonstrate that the efficacy of Smac mimetics is highly contingent upon both drug architecture and tumor-specific molecular contexts. Future directions involve leveraging biomarkers to stratify patient subpopulations most likely to derive clinical benefit, and implementing rational combination regimens to translate these targeted agents into effective tools for overcoming chemoresistance in OC.

#### 2.1.3 Targeting DRs

DRs constitute pivotal initiator molecules within the TNFR superfamily that mediate extrinsic apoptosis. Among these, TRAIL receptors 1 (DR4) and 2 (DR5) can be specifically activated by TNF-related apoptosis-inducing ligand (TRAIL); upon ligand engagement, these receptors facilitate assembly of the death-inducing signaling complex, thereby initiating caspase-8-dependent apoptotic signaling (Bevis et al., 2010). Consequently, recombinant human TRAIL protein and agonistic antibodies targeting DR4/DR5 have demonstrated considerable potential in inducing apoptosis in multiple cancer cell types in preclinical investigations (Yuan et al., 2018). In OC research, diverse compounds have been shown to modulate DR4/DR5 expression through distinct mechanisms, thereby potentiating TRAIL-induced apoptosis. For instance, ONC201 antagonizes dopamine receptor D2, promoting forkhead box O3a (Foxo3a) nuclear translocation and subsequent transcriptional upregulation of TRAIL and DR5, ultimately activating apoptotic signaling (Allen et al., 2013; Fan et al., 2022). Quinacrine operates through a dual-mechanism approach—enhancing DR5 gene transcription while concurrently suppressing its lysosomal degradation—synergistically elevating DR5 protein levels and significantly augmenting OC cell sensitivity to TRAIL (Liang et al., 2020). Additionally, quercetin triggers endoplasmic reticulum stress via reactive oxygen species (ROS) generation, upregulating the C/EBP homologous protein to selectively increase DR5 expression, thereby enhancing apoptotic responses to TRAIL in OC cells and suppressing xenograft growth *in vivo* (Yi et al., 2014). Collectively, these findings underscore that therapeutic strategies targeting DR4/DR5 hold considerable promise for OC treatment.

#### 2.1.4 Targeting the JNK/p38 MAPK pathway

The MAPK family serves as a central signaling hub for cellular stress responses, among which c-Jun N-terminal kinase (JNK) and p38 MAPK constitute two pivotal members. Activated JNK modulates apoptosis-related genes through phosphorylation of transcription factors including c-Jun, whereas p38 MAPK participates broadly in cellular proliferation, differentiation, stress adaptation, and apoptotic processes (Wada et al., 2008). In OC, aberrant activation of the JNK/p38 MAPK pathway is intimately associated with tumor progression, chemoresistance, and unfavorable prognosis (Ma Y. T. et al., 2025), rendering it an important therapeutic target. Studies have demonstrated that sustained p38 MAPK activation constitutes a critical mechanism enabling cancer cells to undergo “anastasis”—a process of recovery from the brink of death—following apoptotic stimulation, thereby acquiring more aggressive phenotypes. Administration of the p38 inhibitor SB203580 during early anastasis effectively abrogates this process, suppressing the migratory and angiogenic capacities of surviving cancer cells (Sun et al., 2023). Additionally, numerous natural compounds exert anti-OC effects through modulation of the JNK/p38 MAPK pathway. For instance, piperine, a natural alkaloid found in the fruit of black and long, induces intrinsic apoptosis in cells via JNK/p38 MAPK activation (Si et al., 2018). Sideroxylin, isolated from *Callistemon lanceolatus*, activates the JNK/p38 MAPK pathway, eliciting mitochondrial dysfunction, reactive oxygen species accumulation, and lipid peroxidation, thereby suppressing cell growth (Park et al., 2018). Tanshinone IIA, conversely, reverses TRAIL resistance in cells by activating p38 MAPK to downregulate survivin expression (Lin et al., 2015). Collectively, the JNK/p38 MAPK pathway exerts a pivotal role in OC progression, therapeutic resistance, and cell fate determination. Consequently, targeting this pathway—particularly through modulation by natural compounds—has emerged as a promising strategy to overcome treatment resistance and suppress malignant phenotypes in OC.

#### 2.1.5 Targeting the PI3K/AKT/mTOR pathway

The PI3K/Akt/mTOR pathway serves as a critical regulatory axis governing cell proliferation, survival, and metabolic homeostasis. Its aberrant activation constitutes a pivotal driver of OC initiation, progression, and chemoresistance, rendering targeted inhibition of this pathway an important therapeutic strategy (Agrawal et al., 2025). Recent studies have revealed that numerous natural products exert anti-OC effects through multi-targeted intervention of this pathway, with mechanistic emphases varying across distinct categories: first, direct inhibition of pivotal nodal points, exemplified by methyl lucidone from *Lindera erythrocarpa* Makino, which suppresses PI3K/Akt/mTOR signaling and coordinately blocks the NF-κB pathway, inducing cell cycle arrest and apoptosis (Yoon et al., 2020); second, activation of upstream negative regulators, as demonstrated by diosgenin-mediated upregulation of the tumor suppressor phosphatase and tensin homolog (PTEN), indirectly inhibiting PI3K/Akt/mTOR signaling to suppress proliferation, migration, and induce apoptosis (Fang et al., 2024); and third, exemplified by isojacareubin derived from *Garcinia nujiangensis*, which simultaneously inhibits the PI3K/Akt/mTOR and MAPK/ERK axes, exhibiting potentiated pro-apoptotic efficacy (Tang et al., 2020). Collectively, these investigations illuminate the therapeutic potential of targeting the PI3K/Akt/mTOR pathway in OC and furnish critical insights for multi-targeted drug discovery based on natural product scaffolds.

#### 2.1.6 Targeting other apoptosis regulators

In recent years, therapeutic target research in OC has expanded beyond the aforementioned canonical pathways, with numerous novel targets being actively explored. For instance, triptonide isolated from *Tripterygium wilfordii* exerts antitumor effects through p38/p53 pathway activation and autophagy-mediated apoptosis induction (Lou et al., 2024). Sodium citrate chelates intracellular Ca^2+^, suppressing the Ca^2+^/CAMKK2/Akt/mTOR signaling axis and subsequently downregulating hypoxia inducible factor 1 subunit alpha-dependent glycolysis to induce apoptosis (Wu et al., 2024). Agrimonolide, a compound derived from *Agrimonia pilosa*, simultaneously induces apoptosis and ferroptosis through targeted stearoyl-CoA desaturase-1 (SCD1) inhibition, thereby suppressing tumor progression (Liu et al., 2022d). Collectively, these studies highlight the therapeutic potential of targeting apoptosis, metabolic reprogramming, and stress signaling in OC, supporting the development of multi-mechanism synergistic anticancer strategies.

### 2.2 Autophagy

**FIGURE 2:**
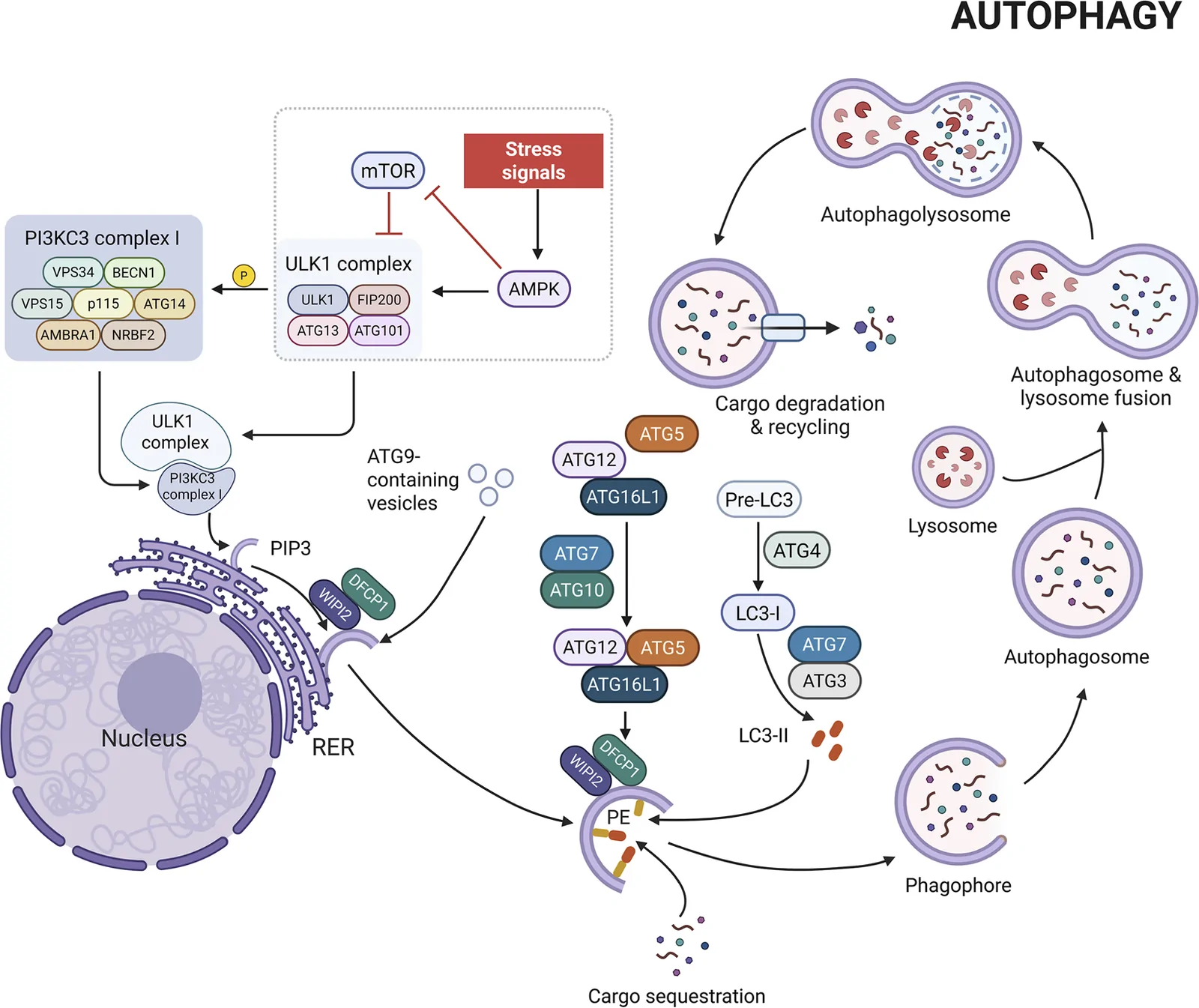
Core autophagic stages in OC: initiation, phagophore nucleation, autophagosome formation, autophagosome-lysosome fusion, and cargo degradation/recycling. Abbreviations: PI3KC3, class III phosphatidylinositol 3-kinase; VPS34, vacuolar protein sorting 34; BECN1, beclin 1; FIP200, focal adhesion kinase family interacting protein of 200 kDa; PIP3, phosphatidylinositol-3,4,5-trisphosphate; RER, rough endoplasmic reticulum; WIPI2, WD repeat domain, phosphoinositide interacting 2; DFCP1, double FYVE-containing protein 1; LC3, microtubule-associated protein 1 light chain 3; PE, phosphatidylethanolamine.

Autophagy represents an evolutionarily conserved intracellular degradation and recycling process wherein cytoplasmic constituents are sequestered within autophagosomes and delivered to lysosomes for degradation, thereby maintaining cellular homeostasis (Figure 2) (Liu S. et al., 2023). This process encompasses multiple sequential stages including initiation, nucleation, elongation, fusion, and degradation, and is precisely orchestrated by molecular machinery comprising the ULK complex, PI3K complex, and autophagy-related (ATG) proteins (Li et al., 2020). Autophagy exerts context-dependent dual roles in tumorigenesis and progression: during early tumorigenesis, autophagy suppresses tumor initiation and progression by eliminating mutant proteins and damaged organelles; conversely, in advanced malignancies, autophagy transforms into a pro-survival system that enables cancer cells to adapt to hypoxia, nutrient deprivation, and chemotherapeutic stress, thereby fostering tumor growth, metastasis, and therapeutic resistance (Onorati et al., 2018). This dynamic duality renders pharmacological modulation of autophagy by small-molecule compounds a highly promising strategy in OC therapeutics.

#### 2.2.1 Targeting the PI3K/AKT/mTOR pathway

The PI3K/Akt/mTOR pathway serves as a critical regulatory node governing both apoptotic execution and autophagic flux, with its aberrant activation intimately linked to invasive growth and malignant progression of tumor cells (Ediriweera et al., 2019). Recent investigations have demonstrated that diverse small-molecule compounds effectively induce autophagic cell death in OC cells through targeted inhibition of this pathway.

##### 2.2.1.1 Synthetic compounds

The dual PI3K/mTOR inhibitor WX390 blocks cytoprotective late-stage autophagic flux through PI3K/Akt/mTOR pathway suppression, thereby eliciting synergistic antitumor effects when combined with cisplatin (Zheng K. et al., 2025). The AMPK activator MK8722 employs a distinctive dual-regulatory mechanism: it initiates early autophagy via PI3K/Akt/mTOR pathway inhibition while concurrently suppressing late autophagy through fatty acid synthase downregulation and disruption of its interaction with the autophagosome-lysosome fusion machinery soluble N-ethylmaleimide-sensitive factor attachment protein receptors (STX17-SNP29-VAMP8). This “prime-and-block” strategy disrupts intact autophagic flux, resulting in toxic autophagic substrate accumulation and tumor growth suppression (Wang L. et al., 2024). Additionally, the novel sirtuin 1 inhibitor MHY2245 induces autophagic cell death and attenuates tumor energy metabolism through concurrent PI3K/Akt/mTOR pathway inhibition and pyruvate kinase M2-mediated glycolysis downregulation, demonstrating antitumor activity in both *in vitro* and *in vivo* models (Tae et al., 2020). Collectively, these investigations demonstrate that targeted intervention of the PI3K/Akt/mTOR signaling axis at distinct nodes, coupled with precise modulation of discrete autophagic stages, effectively disrupts metabolic adaptability and survival homeostasis in OC cells, furnishing critical mechanistic rationale for developing combination therapeutic strategies.

##### 2.2.1.2 Natural compounds

Icariin derived from *Epimedium sagittatum* suppresses autophagy through AKT/mTOR/ATG5 pathway activation, thereby reversing cisplatin resistance in SKVCR cells (Jiang et al., 2019). Conversely, paeonol isolated from *Paeonia suffruticosa* induces cytoprotective autophagy via Akt/mTOR pathway inhibition, and combination with the autophagy inhibitor hydroxychloroquine synergistically enhances its antitumor activity *in vivo* with reduced toxicity relative to cisplatin (Gao et al., 2019). Additionally, daphnetin from Daphne Korean Nakai activates AMPK while suppressing the Akt/mTOR pathway, eliciting ROS-dependent apoptosis and cytoprotective autophagy; co-administration with hydroxychloroquine further potentiates its efficacy (Fan et al., 2021). Collectively, these investigations identify a convergent target for overcoming cisplatin resistance: the PI3K/Akt/mTOR signaling axis and its regulated autophagic processes. Pharmacological targeting of this network by natural products, coupled with synergistic autophagy inhibition, furnishes a clear direction for developing next-generation, low-toxicity chemosensitization regimens.

Targeting the PI3K/Akt/mTOR pathway to modulate autophagy represents a dual-pillar strategy for overcoming cisplatin resistance and enhancing therapeutic efficacy in OC. The aforementioned investigations have validated the feasibility of this approach from diverse perspectives, demonstrating the effectiveness of precise autophagic stage intervention and rationally designed combination regimens. Nevertheless, to surmount the efficacy, toxicity, and resistance bottlenecks confronting clinical translation of these strategies (Ediriweera et al., 2019), future research must prioritize: advancing precision therapeutics based on molecular subtyping, designing intelligent intermittent and combination dosing schedules, and establishing robust biomarker frameworks, thereby facilitating efficient translation from mechanistic insights to clinical benefit.

#### 2.2.2 Targeting ULK1

Unc-51-like autophagy activating kinase 1 (ULK1) serves as a core serine/threonine kinase at the initiation stage of autophagy. It forms a stable complex with FIP200, ATG13, and ATG101, collectively known as the ULK1 complex (Zou et al., 2022). This complex senses cellular nutrient and energy status fluctuations, directly driving early autophagosome formation and activating the downstream Beclin-1 complex to initiate the autophagic cascade (Papinski and Kraft, 2016). Currently, multiple ULK1 inhibitors can enhance immunological killing or induce cell death via autophagy suppression (Zhu et al., 2025). Conversely, the ULK1 activator LYN-1604 (Zhang et al., 2017) suppresses tumor growth and metastasis through promoting ULK1-dependent autophagosome formation. In the context of OC, investigations have demonstrated that ULK1 expression is upregulated and drives autophagic activation in HGSC spheroids. Pharmacological inhibition of ULK1 activity using the small-molecule inhibitor MRT68921 effectively blocks autophagy and markedly attenuates spheroid cell viability. Furthermore, elevated ULK1 expression correlates significantly with unfavorable prognosis in advanced-stage OC patients, suggesting that targeting ULK1 may offer a potential therapeutic strategy for HGSC (Singha et al., 2020). Additionally, the potential of ULK1 in combination therapy has been explored. For instance, combined crizotinib and olaparib treatment enhances ROS levels, suppresses phosphorylation-mediated activation of the AKT/mTOR/ULK1 signaling axis, thereby potentiating autophagy and promoting autophagic cell death. This combination regimen exhibits synergistic antitumor activity across diverse OC models and overcomes autophagy-mediated PARPi resistance (Santiago-O'Farrill et al., 2024). In a separate study, the natural compound cinnamaldehyde derived from *cinnamon* was found to induce mitophagy through ROS-mediated activation of the AMPK/ULK1/Beclin-1 axis, cooperating with apoptotic pathways to suppress OC (Yi et al., 2026). Collectively, ULK1 serves as a critical nexus linking cellular stress to autophagic activation and holds substantial therapeutic value in OC.

#### 2.2.3 Targeting ATG proteins

Autophagosome formation is initiated by phosphatidylinositol 3-phosphate signals generated by vacuolar protein sorting 34, whose core function involves recruiting ATG protein complexes to drive autophagic membrane elongation (Pérez-Hernández et al., 2019). ATG4B serves as the essential protease within this complex, precisely controlling autophagosome expansion and maturation through modulating microtubule-associated protein light chain 3 (LC3) processing and recycling (Yu et al., 2012). Targeting this node, studies have demonstrated that Antrodia salmonea induces ATG4B inhibition via ROS generation, thereby disrupting LC3 cycling, causing LC3-II accumulation, and ultimately triggering autophagic cell death in OC cells (Yang et al., 2019). Notably, while ATG modulators remain understudied in OC, they have been increasingly identified and validated in multiple other malignancies (Fu et al., 2019). In view of this, leveraging existing or developing novel ATG modulators to regulate autophagic activity has emerged as a highly promising translational strategy in OC therapeutics.

#### 2.2.4 Targeting lysosomes

Lysosomes depend on vacuolar ATPase to maintain an intraluminal pH of 4.5–5.5, an acidic milieu essential for activating acid hydrolases and enabling substrate degradation. During autophagy, lysosomes fuse with autophagosomes and release hydrolases to degrade cargo contents, thereby recycling nutrients (Mahapatra et al., 2021). Based on this mechanism, the weakly basic agents chloroquine (CQ) and hydroxychloroquine (HCQ) have been extensively employed as canonical autophagy inhibitors. These compounds diffuse into lysosomes, become protonated and trapped within the acidic milieu, thereby neutralizing acidity, elevating pH, suppressing hydrolase activity, and blocking autophagosome-lysosome fusion and subsequent degradation, ultimately exerting autophagy-inhibitory effects (Ferreira et al., 2021). However, chronic administration of CQ and HCQ carries significant risks of dose-cumulative toxicities, including retinopathy and cardiotoxicity, with inconsistent clinical efficacy (Melles and Marmor, 2014). Consequently, research priorities have shifted toward developing novel autophagy inhibitors with enhanced potency and reduced toxicity (Rebecca et al., 2017), aiming to circumvent the limitations of conventional agents. In OC research, diverse novel inhibitors have demonstrated distinctive mechanisms and therapeutic potential. For instance, elaiophylin disrupts lysosomal function—encompassing cathepsin inhibition and membrane permeabilization—to block autophagic flux, exhibiting a favorable therapeutic window with efficacy at low doses and gastrointestinal toxicity manifesting only at high doses (Zhao et al., 2015). Dihydrotanshinone I targets sortilin 1 for degradation, alleviating its suppression of ATG5/ATG16L1; this promotes autophagosome formation while concurrently blocking fusion with lysosomes, thereby inducing autophagic cell death (Sun et al., 2025). Costunolide, through dual inhibition of autophagy initiation (via AMPK/mTOR pathway blockade) and autophagosome-lysosome fusion (via disruption of the syntaxin 17–synaptosomeassociated protein 29–vesicle associated membrane protein 8 complex), synergistically arrests autophagic flux and enhances cisplatin chemosensitivity in OC cells (Liang et al., 2023). Collectively, these novel autophagy inhibitors, acting upon distinct molecular targets and autophagic stages, not only illuminate the intricate regulatory network of autophagy but also furnish new directions for circumventing the toxicity and efficacy limitations of conventional agents, holding substantial promise for more selective strategies in combination therapy and chemoresistance reversal for OC.

#### 2.2.5 Targeting other autophagy regulators

In recent years, research targets for autophagy modulation in OC have undergone continuous expansion, extending from canonical pathways to a multi-tiered system encompassing pivotal kinases, epigenetic regulators, and organelle functional proteins. At the kinase level, focal adhesion kinase (FAK) governs not only cancer cell adhesion and migration but also autophagic processes through downstream Src/Akt signaling; its novel inhibitor E2 induces cytotoxic autophagy in OC cells, suppressing tumor growth and metastasis *in vivo* and furnishing experimental rationale for FAK-targeted autophagy modulation (Feng et al., 2024). Regarding transcriptional and epigenetic regulation, targeting lncRNA has emerged as a critical strategy. For instance, berberine disrupts the interaction between lncRNA LINC00123 and transcription factor p65, promoting p65 degradation and suppressing MAPK10 expression, thereby coordinately inhibiting autophagy and glycolysis and demonstrating antitumor potential in preclinical models (Yan et al., 2024). At the organelle functional level, Sulfur heteroarotinoid A2 (SHetA2) binds directly to the substrate-binding domain of mortalin, inhibiting its mitochondrial localization and consequently activating PINK1/Parkin-mediated mitophagy selectively in OC cells to induce cell death, whereas normal cells resist this cytotoxicity through maintained mitochondrial fusion capacity (Chandra et al., 2025). Collectively, multi-layered autophagy modulation in OC through targeting kinases, epigenetic regulators, and organelle functional proteins not only deepens understanding of tumor cell fate determination mechanisms but also furnishes novel directions for developing precise and synergistic therapeutic strategies.

### 2.3 Necroptosis

**FIGURE 3:**
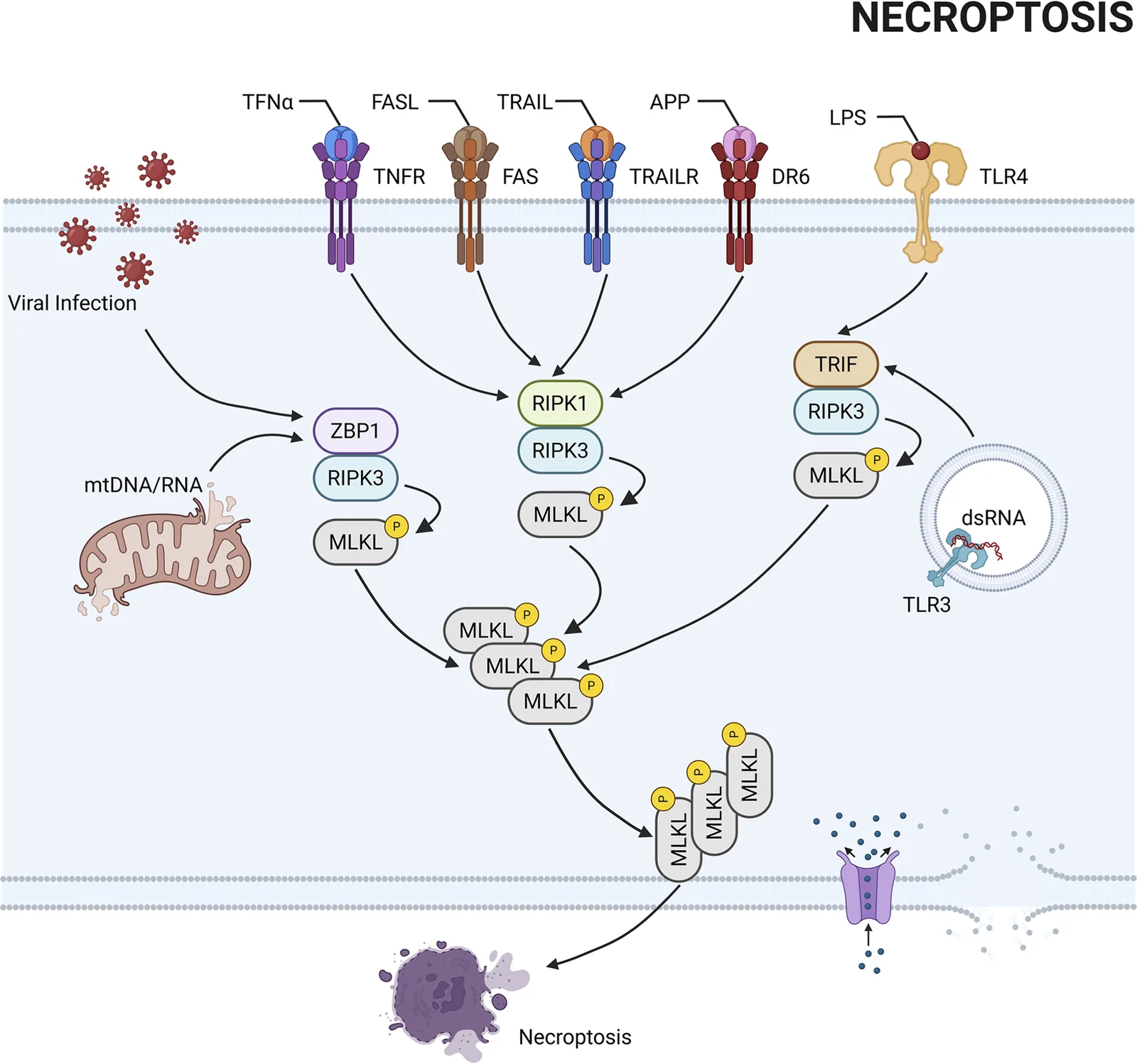
Necroptotic signaling in OC. RIPK3-mediated MLKL phosphorylation triggers oligomerization and membrane translocation, leading to pore formation, membrane rupture, and inflammatory cell death. Abbreviations: FASL, Fas ligand; FAS, Fas receptor; TRAILR, TRAIL receptor; APP, amyloid precursor protein; LPS, lipopolysaccharide; TLR, Toll-like receptor; TRIF, TIR-domain-containing adapter-inducing interferon-β; ZBP1, Z-DNA binding protein 1; mtDNA/RNA, mitochondrial DNA/RNA; dsRNA, double-stranded RNA.

Necroptosis represents a regulated form of cell death characterized by pronounced swelling of cells and organelles, loss of plasma membrane integrity, and release of intracellular contents, consequently eliciting lytic changes and localized inflammatory responses (Figure 3) (Vandenabeele et al., 2010). This process is orchestrated through the coordinated activity of receptor-interacting serine/threonine-protein kinase 1 (RIPK1), RIPK3, and mixed lineage kinase domain-like protein (MLKL). Upon apoptotic blockade, RIPK1 engages with RIPK3 to activate MLKL, which subsequently forms pores on the plasma membrane, culminating in cell lysis (Martens et al., 2021). In cancer therapeutics, necroptosis exhibits a dichotomous nature: on one hand, it enhances antitumor immunity through induction of immunogenic cell death, thereby suppressing tumor growth (Gong et al., 2019); conversely, the inflammatory responses triggered may foster tumor progression (Seifert et al., 2021). Currently, diverse chemotherapeutic agents and natural compounds have been validated to potentiate antitumor efficacy through necroptosis induction (Qin et al., 2019). Consequently, modulation of necroptotic signaling has emerged as a novel therapeutic strategy in oncology.

#### 2.3.1 Targeting the RIPK1/RIPK3/MLKL pathway

Analysis of over 60 cancer cell lines revealed that approximately two-thirds of samples exhibit silenced RIPK3 expression and consequent loss of RIPK3 function, resulting in impaired chemotherapy-induced MLKL phosphorylation and marked suppression of downstream necroptotic signaling, suggesting that RIPK3 deficiency confers survival advantages upon cancer cells (Koo et al., 2015). Clinical investigations have further validated the prognostic value of this pathway: high RIPK3 expression in OC tissue correlates significantly with prolonged progression-free survival (Zheng M. et al., 2025); the RIPK1 genetic single-nucleotide polymorphism (SNP) rs9392453 is associated with epithelial OC susceptibility, whereas rs6907943 relates to patient survival, with the AC genotype serving as an independent protective factor in early-onset epithelial OC patients (Wang et al., 2023); additionally, downregulated MLKL expression is significantly associated with shortened overall and disease-free survival in patients with resectable primary OC (He et al., 2013). Consequently, given the pivotal role of the RIPK1/RIPK3/MLKL pathway in OC pathogenesis, progression, and prognostic stratification, targeted induction of necroptosis has emerged as a highly promising therapeutic strategy. Currently, small-molecule compounds targeting necroptotic pathways are emerging continuously, demonstrating potential antitumor value. For instance, the BMI1 inhibitor PTC-209 induces adenosine triphosphate depletion in OC cells, triggering autophagy and subsequently activating the PINK1-PARK2-mediated mitophagy pathway, thereby initiating RIPK1-RIPK3-MLKL signaling and ultimately executing necroptosis (Dey et al., 2016). Sulfasalazine upregulates p62/sequestosome-1 in cisplatin-resistant SKOV3/DDP cells, which, dependent on its zinc finger domain serving as a scaffold protein, facilitates formation of RIPK1-RIPK3-MLKL necrosome complex, thereby inducing necroptosis and overcoming apoptotic resistance (Liu N. et al., 2024). The natural flavonoid fisetin induces apoptosis in OC cells while concurrently triggering necroptosis through the Z-DNA binding protein 1-mediated RIPK3-MLKL signaling pathway (Liu et al., 2022c). Recently, Li et al. reported a biomimetic chitosan nanogel platform for co-delivery of gambogic acid (GA) and MAS1 small activating RNA (saMAS1). GA induces necroptosis in OC cells through caspase-8 inhibition and RIPK1/RIPK3/MLKL pathway activation, whereas saMAS1 suppresses tumor metastasis via MAS1 upregulation. This platform, combined with cell membrane biomimetic targeted delivery, achieves synergistic therapeutic efficacy against both primary tumor growth and metastasis in OC (Li et al., 2025). Collectively, although diverse compounds capable of inducing necroptosis have been identified, their clinical utility and selectivity in OC therapeutics remain to be validated. Emerging nanodelivery technologies offer novel opportunities for enhancing selective tumor cell killing through nanocarrier-mediated delivery of necroptosis inducers, effectively addressing apoptotic resistance in OC.

### 2.4 Pyroptosis

**FIGURE 4:**
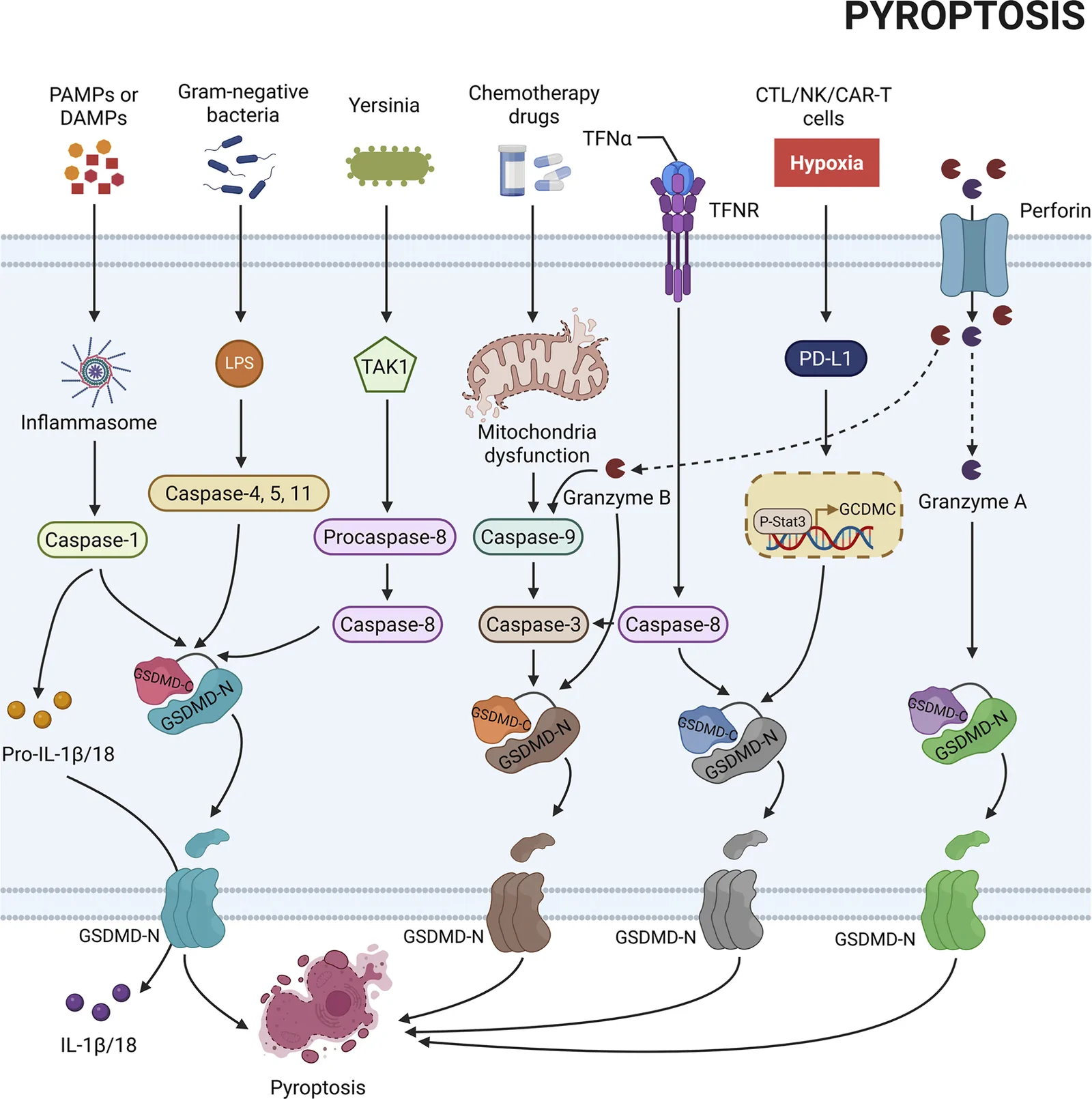
Pyroptotic execution in OC. Diverse stimuli activate GSDM proteins via canonical/non-canonical inflammasomes, caspase-3/8, or granzyme pathways, leading to membrane pore formation, cell lysis, and inflammatory cytokine release. Abbreviations: PAMPs, pathogen-associated molecular patterns; DAMPs, damage-associated molecular patterns; TAK1, TGF-β-activated kinase 1; CTL, cytotoxic T lymphocyte; NK, natural killer cell; CAR-T, chimeric antigen receptor T-cell; PD-L1, programmed death-ligand 1.

Pyroptosis represents a regulated form of cell death characterized by cellular lysis, inflammatory responses, cell swelling, chromatin fragmentation, plasma membrane perforation, and release of pro-inflammatory cytokines including interleukin-1beta (IL-1β) and interleukin-18 (IL-18) (Figure 4) (Martinon et al., 2002). This process is typically triggered by inflammasome-activated caspases, with gasdermin (GSDM) family proteins—particularly GSDMD—serving as the core executioners. The N-terminal domain of GSDMD forms pores on the plasma membrane, culminating in osmotic lysis (Shi et al., 2017). Pyroptotic regulation encompasses diverse pathways, including the canonical caspase-1-dependent inflammasome pathway, the non-canonical caspase-4/5/11-dependent pathway, and caspase-3/8-mediated as well as granzyme-mediated routes (Wang and Wan, 2023). In oncology, pyroptosis exhibits a dichotomous nature (Du et al., 2021): robust pyroptosis effectively induces cancer cell death and activates antitumor immune responses, thereby suppressing tumor growth; conversely, attenuated pyroptosis may exacerbate inflammatory reactions within the tumor microenvironment, paradoxically fostering tumor progression and metastasis. This duality renders pyroptosis a highly promising therapeutic target in cancer. Recent investigations have demonstrated that targeting pyroptotic pathways in OC holds potential for improving therapeutic outcomes, emerging as a novel direction for future treatment strategies.

#### 2.4.1 Targeting GSDM

The GSDM family proteins constitute the pivotal executioners of pyroptosis. This family comprises six members in humans: GSDMA, GSDMB, GSDMC, GSDMD, GSDME, and DFNB59 (Pejvakin) (Yang et al., 2022). With the exception of DFNB59, all GSDM proteins consist of an N-terminal domain (GSDM-NT) with pore-forming activity and a C-terminal domain (GSDM-CT) with autoinhibitory function, connected by a linker peptide. Under resting conditions, GSDM-CT binds to and suppresses GSDM-NT activity; upon reception of specific death or inflammatory signals, the linker peptide is cleaved, releasing GSDM-NT. GSDM-NT subsequently translocates to the plasma membrane and oligomerizes to form pores, ultimately driving pyroptosis (Orning et al., 2019). Consequently, the GSDM family is recognized as the central executioner of pyroptosis. Among these, GSDMD and GSDME are the most extensively studied proteins, playing critical roles in the inflammasome pathway and chemotherapy-induced cell death, respectively (Wang and Wan, 2023). In recent years, accumulating evidence has demonstrated that GSDM family members hold substantial therapeutic potential in OC. For instance, the polo-like kinase 1 inhibitor BI 2536 triggers pyroptosis via caspase-3/GSDME pathway activation, concurrently promoting CD8^+^ T cell infiltration at tumor sites and furnishing novel insights for activating antitumor immune responses (Huo et al., 2022). Nobiletin, derived from citrus, induces mitochondrial membrane potential dissipation and ROS accumulation, ultimately eliciting GSDMD/GSDME-dependent pyroptosis through autophagy modulation (Zhang et al., 2020). α-NETA activates the GSDMD/caspase-4 pathway to trigger pyroptosis in epithelial OC cells, characterized by plasma membrane blebbing and IL-18 release, and significantly suppresses tumor growth in both *in vitro* and *in vivo* models (Qiao et al., 2019). Notably, GSDMA is upregulated in OC tissues, with elevated expression predicting unfavorable prognosis and serving as a potential prognostic biomarker (Liu T. et al., 2022). Collectively, pharmacological modulation of GSDM proteins has emerged as a significant therapeutic option for OC. Future integration of computer-aided drug design technologies (Uba et al., 2024), through precise structural modeling of target proteins, will enable virtual screening and molecular docking to predict binding modes and affinities of small-molecule candidates, thereby guiding lead compound optimization and furnishing novel opportunities for pyroptosis-based therapeutic strategies against OC.

#### 2.4.2 Targeting inflammasomes

Inflammasomes are cytoplasmic multiprotein complexes that serve as core components of the innate immune system, sensing pathogenic or cellular damage signals and promoting maturation of pro-inflammatory cytokines including IL-1β through caspase-1 activation, thereby inducing GSDMD-dependent pyroptosis (Rathinam and Fitzgerald, 2016). Recent investigations have demonstrated that inflammasomes play pivotal roles in OC initiation and progression. Inflammasome-associated molecules including IL-1β are markedly upregulated in OC tissues and intimately associated with unfavorable patient prognosis (Liu C. et al., 2022), suggesting that aberrant activation may drive tumorigenesis. Substantial advances have been achieved regarding inflammasome regulatory mechanisms and clinical implications. On one hand, inflammasome-related molecules participate in regulating malignant behaviors of OC cells. Wu et al. demonstrated that microRNA-22 suppresses OC cell proliferation and epithelial-mesenchymal transition through targeted nucleotide-binding domain, leucine-rich-containing family, pyrin domain-containing-3 (NLRP3) inhibition and PI3K/Akt pathway modulation (Wu et al., 2021). On the other hand, inflammasome signaling influences therapeutic responses in OC. Hsu et al. reported that elevated absent in melanoma 2 expression correlates with bevacizumab resistance in epithelial OC patients, serving as a biomarker for predicting anti-angiogenic therapy efficacy (Hsu et al., 2021). Collectively, these findings indicate that inflammasomes constitute not only drivers of OC progression but also critical modulators of treatment response. Based on these discoveries, targeting inflammasomes and their effector pathways has emerged as a novel research direction in OC therapeutics. Multiple intervention strategies have been explored. For instance, statins induce pyroptosis in AT-rich interaction domain 1A-mutant OC cells through mevalonate pathway inhibition, reduced geranylgeranyl pyrophosphate synthesis, impaired Ras homolog family member A (RhoA) prenylation, and subsequent NLRP3/caspase-1/GSDMD axis activation, furnishing potential therapeutic strategies for this genomically defined OC subtype (Zhou et al., 2023). Additionally, Stojanovic et al. demonstrated that DNA methyltransferase inhibitor azacytidine combined with PARPi talazoparib upregulates zinc finger-containing 1, induces mitochondrial DNA leakage into the cytoplasm, and triggers stimulator of interferon genes-dependent inflammasome signaling, exerting tumor-suppressive functions in HGSC (Stojanovic et al., 2025). Although research on inflammasome targeting in OC remains at an early stage, these advances underscore its substantial potential as a therapeutic target. Comprehensive understanding of inflammasome regulatory mechanisms and their complex roles within the OC microenvironment promises to establish foundations for developing more effective and precise therapeutic regimens.

## 3 Metal-dependent cell death

### 3.1 Ferroptosis

**FIGURE 5:**
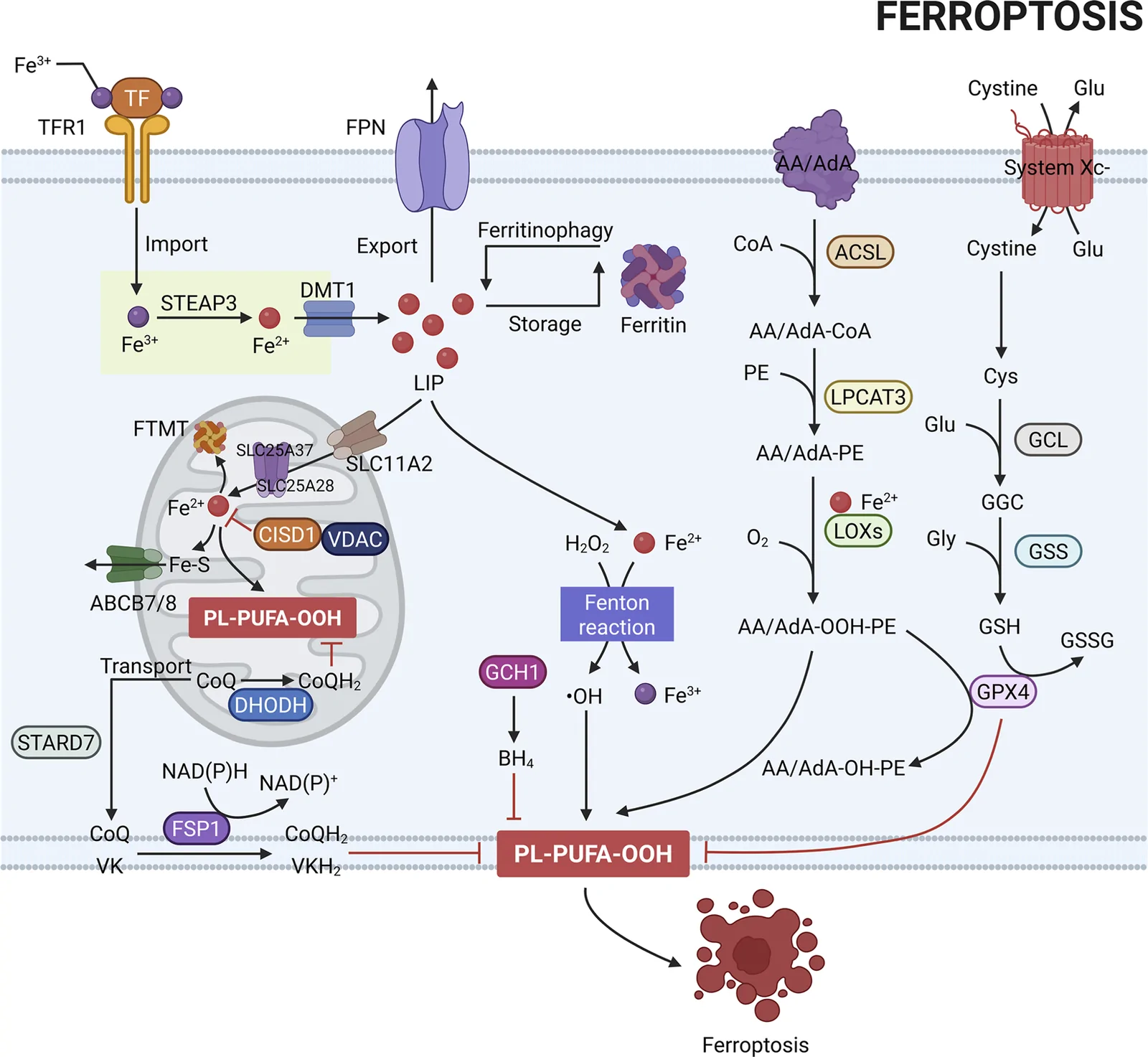
Ferroptosis regulation in OC. Pro-ferroptotic drivers (lipid peroxidation, amino acid metabolic dysregulation, iron overload) overwhelm the GPX4–GSH, FSP1–CoQH_2_, DHODH–CoQH_2_, and GCH1–BH_4_ defense systems, resulting in lethal lipid peroxide accumulation and membrane rupture. Abbreviations: TF, transferrin; TFR1, transferrin receptor 1; FPN, ferroportin; DMT1, divalent metal transporter 1; STEAP3, six-transmembrane epithelial antigen of the prostate 3; FTMT, mitochondrial ferritin; CISD1, CDGSH iron sulfur domain 1; VDAC, voltage-dependent anion channel; ABCB7/8, ATP-binding cassette subfamily B member 7/8; FSP1, ferroptosis suppressor protein 1; STARD7, START domain containing 7; VK, vitamin K; VKH_2_, hydroquinone vitamin K; ACSL, acyl-CoA synthetase long-chain; LPCAT3, lysophosphatidylcholine acyltransferase 3; LOXs, lipoxygenases; GCL, glutamate-cysteine ligase; GSS, glutathione synthetase; GSH, glutathione; GSSG, oxidized glutathione; GCH1, GTP cyclohydrolase 1; BH_4_, tetrahydrobiopterin; PL-PUFA-OOH, phospholipid polyunsaturated fatty acid hydroperoxide.

Ferroptosis represents an iron-dependent form of RCD driven by the accumulation of lipid peroxides, with its most distinctive morphological features localized to mitochondria, manifested as reduced volume, increased membrane density, and diminished or absent cristae (Figure 5) (Dixon et al., 2012). The regulation of this process involves a complex, multi-pathway network encompassing intracellular iron metabolism, lipid metabolism, glutathione (GSH) metabolism, and diverse antioxidant defense systems; the dynamic equilibrium among these pathways collectively governs cellular susceptibility to ferroptosis (Dixon and Olzmann, 2024). Cancer cells, for instance, frequently evade ferroptosis through multiple adaptive mechanisms to acquire survival advantages. These pivotal strategies include: restricting labile iron pool levels, suppressing synthesis and peroxidation of polyunsaturated fatty acid-containing phospholipids, and enhancing endogenous antioxidant defense capacity centered on GSH peroxidase 4 (GPX4) (Hangauer et al., 2017). The synergistic interplay of these mechanisms constitutes a critical foundation for cancer cell resistance to ferroptosis induction. Targeting these resistance mechanisms, anticancer drug development aimed at inducing ferroptosis in cancer cells has been extensively pursued in recent years. Consequently, inducing ferroptosis in OC cells has emerged as a potential therapeutic avenue for improving clinical outcomes and offering renewed hope for patients.

#### 3.1.1 Targeting GPX4

GPX4 serves as a pivotal intracellular antioxidant enzyme that suppresses lipid peroxidation and maintains redox homeostasis through GSH-dependent scavenging of lipid hydroperoxides (Liu Y. et al., 2023). Investigations have revealed that cancer cells with high mesenchymal phenotypes, particularly those exhibiting therapy resistance, exhibit lipid metabolic reprogramming that renders their survival critically dependent upon GPX4 function (Viswanathan et al., 2017). Consequently, targeted GPX4 inhibition to selectively induce ferroptosis in such populations is recognized as an effective strategy for eradicating resistant cancer cell subsets. Numerous compounds have been validated as GPX4 inhibitors capable of inducing ferroptosis (Li B. et al., 2024). For OC exhibiting ferroptosis resistance due to GPX4 accumulation secondary to BRCA1 deficiency, combined ML210 and PARPi olaparib treatment elicits synergistic lethality (Xie et al., 2024). Furthermore, shikonin synergizes with cisplatin to induce ferroptosis through heme oxygenase 1-mediated Fe^2+^ accumulation and GPX4 downregulation (Ni et al., 2023); the natural compound isoginkgetin functions as a novel ubiquitin-specific protease 8 inhibitor that triggers ferroptosis by blocking USP8-mediated deubiquitination of GPX4, thereby promoting its proteasomal degradation (Hu B. et al., 2025). Collectively, these investigations establish that targeting GPX4—whether through direct inhibition, targeted degradation, or coordinated modulation of upstream regulatory pathways—to induce ferroptosis constitutes a highly promising strategy for overcoming chemoresistance and enhancing therapeutic efficacy in OC.

#### 3.1.2 Targeting DHODH

Dihydroorotate dehydrogenase (DHODH) serves as a multifunctional enzyme that regulates *de novo* pyrimidine synthesis, mitochondrial metabolism, and ferroptosis defense, constituting a metabolic vulnerability target in cancer (Lin F. et al., 2025). In ferroptosis defense, DHODH exerts protective effects through the coenzyme Q (CoQ)/reduced CoQ (CoQH_2_) cycle: while catalyzing the oxidation of dihydroorotate to orotate, it concomitantly reduces CoQ to CoQH_2_, thereby leveraging the lipophilic antioxidant activity of CoQH_2_ to scavenge lipid radicals and suppress lipid peroxidation and ferroptosis (Mao et al., 2021). In ovarian clear cell carcinoma, the microtubule inhibitor eribulin downregulates DHODH expression, suppresses the NRF2-heme oxygenase-1 pathway, and diminishes superoxide dismutase activity, synergistically promoting lipid peroxidation and mitochondrial dysfunction to ultimately induce ferroptosis (Azumi et al., 2024). In platinum-resistant OC, DHODH-mediated metabolic reprogramming of pyrimidine synthesis and associated mitochondrial dysfunction—such as reduced spare respiratory capacity—constitutes a distinctive metabolic vulnerability (Cardenas et al., 2026). Although this study did not directly focus on ferroptosis, the revealed mitochondrial homeostasis imbalance and redox stress provide crucial mechanistic insights into the potential role of DHODH in ferroptosis regulation. Collectively, DHODH plays a pivotal role in metabolic regulation and cell death modulation in OC; targeting DHODH may offer novel therapeutic strategies through disrupting pyrimidine metabolism, perturbing mitochondrial homeostasis, and inducing ferroptosis.

#### 3.1.3 Targeting SLC7A11

Solute carrier family 7 member 11 (SLC7A11) serves as the catalytic subunit of the system xc(−) cystine/glutamate antiporter. It mediates the electroneutral exchange of extracellular cystine for intracellular glutamate at a 1:1 stoichiometric ratio, thereby facilitating intracellular cystine reduction to cysteine to support GSH synthesis, enhancing cellular antioxidant defense, and suppressing ferroptosis (Koppula et al., 2021). In OC, elevated co-expression of SLC7A11 and GPX4 has been identified as an independent risk factor for platinum resistance and poor prognosis (Fantone et al., 2024). Notably, high SLC7A11 expression creates a “metabolic double-edged sword”: while it strengthens antioxidant capacity and promotes tumor cell survival, it also increases cancer cell dependence on glucose metabolism. Under glucose deprivation, nicotinamide adenine dinucleotide phosphate (NADPH) depletion induces metabolic crisis, and this vulnerability represents a potential target for therapeutic intervention (Su Z. et al., 2025). Accumulating evidence demonstrates that various compounds induce ferroptosis in OC cells by targeting SLC7A11, showing significant antitumor effects. For example, Icariside II downregulates SLC7A11 to trigger ferroptosis (Yuan et al., 2025). Lidocaine induces ferroptosis via the microRNA-382-5p/SLC7A11 axis (Sun et al., 2021). Additionally, artesunate specifically binds to lysine 250 of glycoprotein 130 (GP130), disrupting IL-6/IL-6 receptor/GP130 complex formation. This inhibits STAT3 phosphorylation and subsequent OTU domain-containing ubiquitin aldehyde-binding protein 1 (OTUB1) transcription, resulting in reduced OTUB1-mediated deubiquitination of SLC7A11, ultimately leading to SLC7A11 ubiquitination and degradation, and consequent ferroptosis induction (Nie et al., 2025). Despite the promising therapeutic prospects of SLC7A11-targeted strategies in OC, clinical translation faces multiple challenges, including inhibitor efficacy, bioavailability, and selectivity (Koppula et al., 2021). Future research should further elucidate tumor metabolic heterogeneity and resistance mechanisms, and integrate multidisciplinary approaches such as nanodelivery systems and combination therapeutic strategies to facilitate the clinical application of this target (Liang et al., 2019).

#### 3.1.4 Targeting GSH

GSH serves as a pivotal intracellular antioxidant that plays a dual and opposing role in cancer initiation and progression. On one hand, it exerts cancer-preventive effects through GSH-s-transferase mediated detoxification of carcinogens; on the other hand, in established tumors, cancer cells frequently upregulate GSH levels to adapt to heightened oxidative stress, thereby promoting tumor survival, proliferation, and contributing to chemotherapy resistance (Chang et al., 2025). Consequently, targeting GSH synthesis or metabolic pathways to attenuate its cytoprotective effects has emerged as a potential strategy to enhance tumor sensitivity to conventional therapies including chemotherapy and radiotherapy (Bansal and Simon, 2018). In OC research, multiple experimental studies have demonstrated that modulation of GSH-related pathways can effectively reverse cisplatin resistance. For instance, Theaflavin-3,3′-digallate (TF3) enhances cisplatin accumulation and DNA damage in OC cells through GSH downregulation and copper transporter 1 upregulation, offering a potential adjuvant strategy for resistance reversal (Pan et al., 2018). Daphnetin induces GSH system imbalance through NAD(P)H quinone oxidoreductase 1 (NQO1) inhibition, manifested by decreased GSH/oxidized GSH ratio and reduced expression of SLC7A11 and GPX4, subsequently triggering iron accumulation and lipid peroxidation, ultimately leading to ferroptosis and enhanced cisplatin chemosensitivity (Ma et al., 2024). Furthermore, combined application of Paired-Box 8 (PAX8) inhibitors (such as losartan) with RSL3 synergistically induces ferroptosis and suppresses tumor growth in OC cells through inhibition of the PAX8-glutamate-cysteine ligase catalytic subunit pathway and consequent downregulation of GSH synthesis (Luo et al., 2024). Collectively, these findings demonstrate that disrupting cancer cell redox homeostasis through targeting GSH metabolism represents an effective approach for chemotherapy sensitization and resistance reversal, providing novel combination therapeutic strategies and experimental evidence for clinical management of OC.

#### 3.1.5 Targeting other ferroptosis regulators

In recent years, beyond the aforementioned classical targets, a series of novel ferroptosis regulatory molecules have emerged as research hotspots for OC therapy. For example, the SPHK1 inhibitor PF-543 reverses olaparib resistance by blocking the SPHK1/NF-κB/NRF2 signaling axis, thereby relieving NRF2-mediated transcriptional repression of ferroptosis-related genes (Teng et al., 2025). NTX-301 upregulates sirtuin 1 transcription through DNA demethylation, subsequently suppressing SCD activity, disrupting lipid homeostasis, and inducing ferroptosis (Wang Y. et al., 2024). Additionally, the novel small-molecule conjugate linoleic acid and glucosamine hybrid (LA–GlcN) targets glucose transporter 1 (GLUT1) to enter HGSC cells, exploiting their intrinsic high-iron environment to selectively induce ferroptosis (Chen et al., 2022). Collectively, these studies reveal the therapeutic potential of targeting distinct regulatory nodes of ferroptosis in OC, providing new directions for overcoming drug resistance and achieving selective cancer cell elimination.

### 3.2 Cuproptosis

**FIGURE 6:**
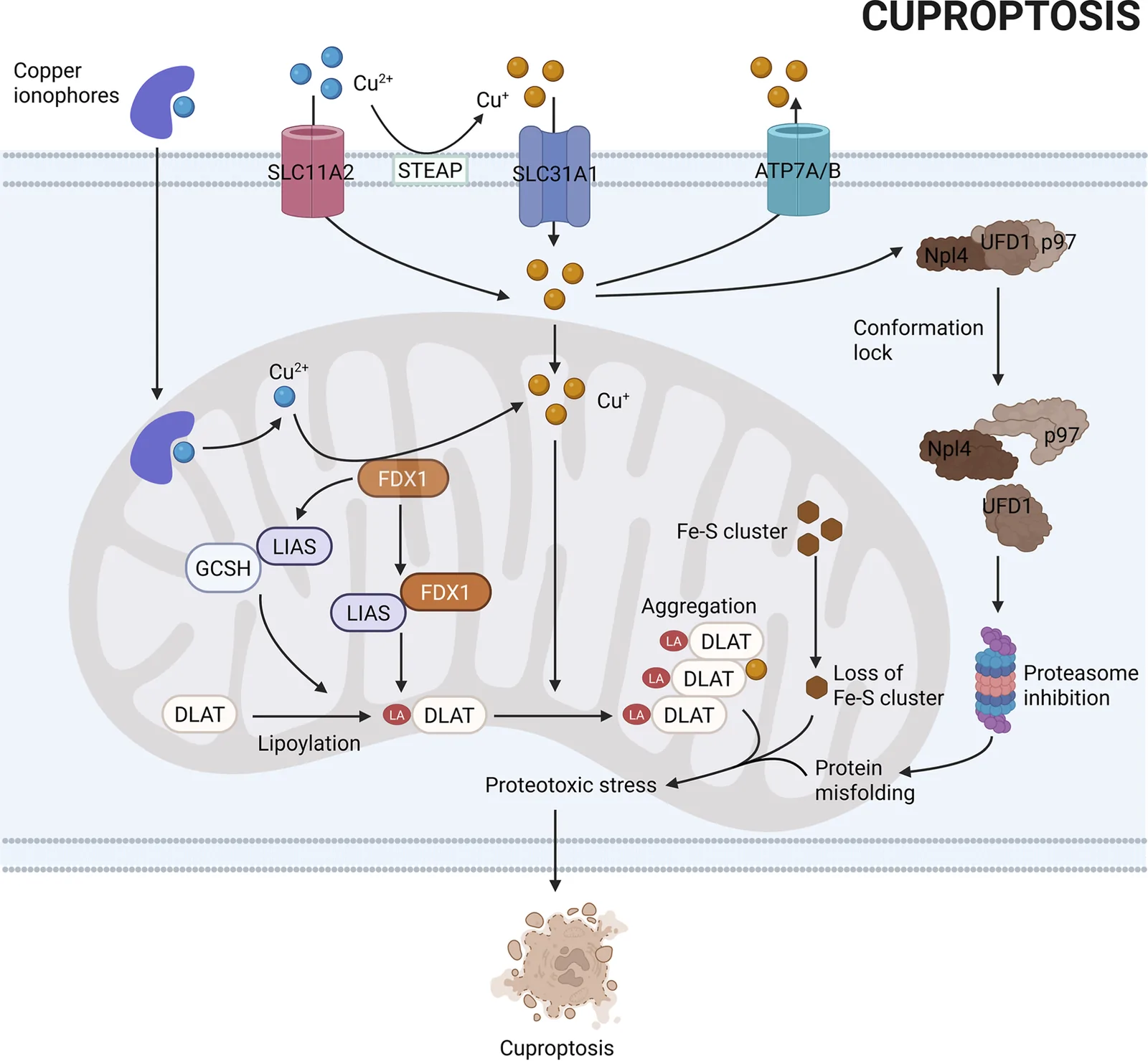
Dual routes to cuproptosis in OC. FDX1-driven Cu^2+^/Cu^+^ reduction triggers mitochondrial protein lipoylation, DLAT aggregation, and Fe-S destabilization (mitochondrial route); cytoplasmic copper ionophores disrupt Npl4-p97 and ubiquitin-proteasome function (proteasome route). Both converge on proteostasis collapse. Abbreviations: SLC31A1, solute carrier family 31 member 1; ATP7A/B, ATPase copper transporting alpha/beta; LIAS, lipoic acid synthetase; GCSH, glycine cleavage system protein H; LA, lipoic acid; Fe-S, iron-sulfur; Npl4, nuclear protein localization 4; UFD1, ubiquitin recognition factor in ER associated degradation 1; p97, valosin-containing protein.

Cuproptosis is a distinct form of RCD induced by intracellular copper overload, with its core mechanism involving proteotoxic stress responses (Figure 6). Tsvetkov and colleagues first observed this phenomenon in 2019 and formally established it in 2022 (Tsvetkov et al., 2022). The occurrence of cuproptosis is closely associated with mitochondrial function. In the mitochondria-dependent pathway, excess copper ions are reduced by ferredoxin 1 (FDX1) and subsequently participate in two critical processes: binding to lipoic acid synthase (LIAS) to promote protein lipoylation, and directly interacting with lipoylated substrates such as dihydrolipoamide S-acetyltransferase (DLAT), leading to aberrant aggregation of these proteins. This aggregation generates toxicity, disrupts iron-sulfur cluster protein stability, and ultimately triggers proteotoxic stress and heat shock responses, thereby selectively eliminating tumor cells with high oxidative phosphorylation activity and abundant lipoylated substrates (Lin C. H. et al., 2024). Additionally, mitochondria-independent pathways exist, including cytoplasmic p97-Npl4 complex aggregation and inhibition of the ubiquitin-proteasome system (Meng et al., 2025). Notably, cellular metabolic status influences sensitivity to cuproptosis: enhanced tricarboxylic acid cycle activity promotes its occurrence, whereas high glycolytic status suppresses it (Meng et al., 2025). Given its unique mechanism, cuproptosis has emerged as a novel potential target for cancer therapy. Strategies under exploration include copper ionophores (Zhao J. et al., 2025), small-molecule drugs targeting key proteins (Tao et al., 2025), and nanoformulations (Wu et al., 2019).

#### 3.2.1 Targeting FDX1

FDX1 is encoded by adjacent genes in the human chromosome 11q22 region and functions as a mitochondrial iron-sulfur protein. Serving as a specific electron donor, it provides reducing equivalents for various mitochondrial cytochrome P450 enzymes, thereby participating in the biosynthesis of steroid hormones, bioactive vitamin D, and bile acids (Lu et al., 2025). Recent studies have identified FDX1 as a critical regulator of cuproptosis with dual functional roles: first, it enhances copper toxicity by reducing Cu^2+^ to Cu^+^; second, it binds to LIAS as an upstream regulator of lipoylation modification, modulating mitochondrial enzyme lipoylation and consequently mediating cuproptosis (Wu A. et al., 2025). Analysis of The Cancer Genome Atlas database and immunohistochemical detection revealed significantly elevated FDX1 protein levels in OC tissues (Liu C. et al., 2025), suggesting its potential in promoting tumorigenesis and progression. Elesclomol has been demonstrated to inhibit tumor growth through FDX1-mediated cuproptosis pathways and enhance cisplatin efficacy (Zou et al., 2025). Its mechanism involves targeting mitochondrial complex IV, thereby regulating FDX1 expression through LRPPRC (Wu Y. et al., 2025). Disulfiram, originally developed as an alcohol-aversion drug, induces apoptosis through the Bax/Bcl-2/caspase-3 signaling pathway when used alone. However, when combined with copper to form the diethyldithiocarbamate-copper complex, it downregulates FDX1 expression and disrupts Fe-S cluster protein stability, thereby inducing cuproptosis; this combination efficacy has been validated in animal experiments (Gan et al., 2023). Additionally, anisomycin, a protein synthesis inhibitor that binds to the 60S ribosomal subunit, significantly suppresses transcription factor yin-yang 1 expression and downregulates transcription of key lipoyl metabolism genes including FDX1 and DLAT, ultimately inducing cuproptosis in OC stem cells (Nie et al., 2022). Collectively, these findings demonstrate that FDX1 is highly expressed in OC with both pro-tumorigenic potential and therapeutic value. Targeting FDX1-mediated cuproptosis provides a novel strategy for OC treatment.

## 4 Other cell death

### 4.1 Parthanatos

**FIGURE 7:**
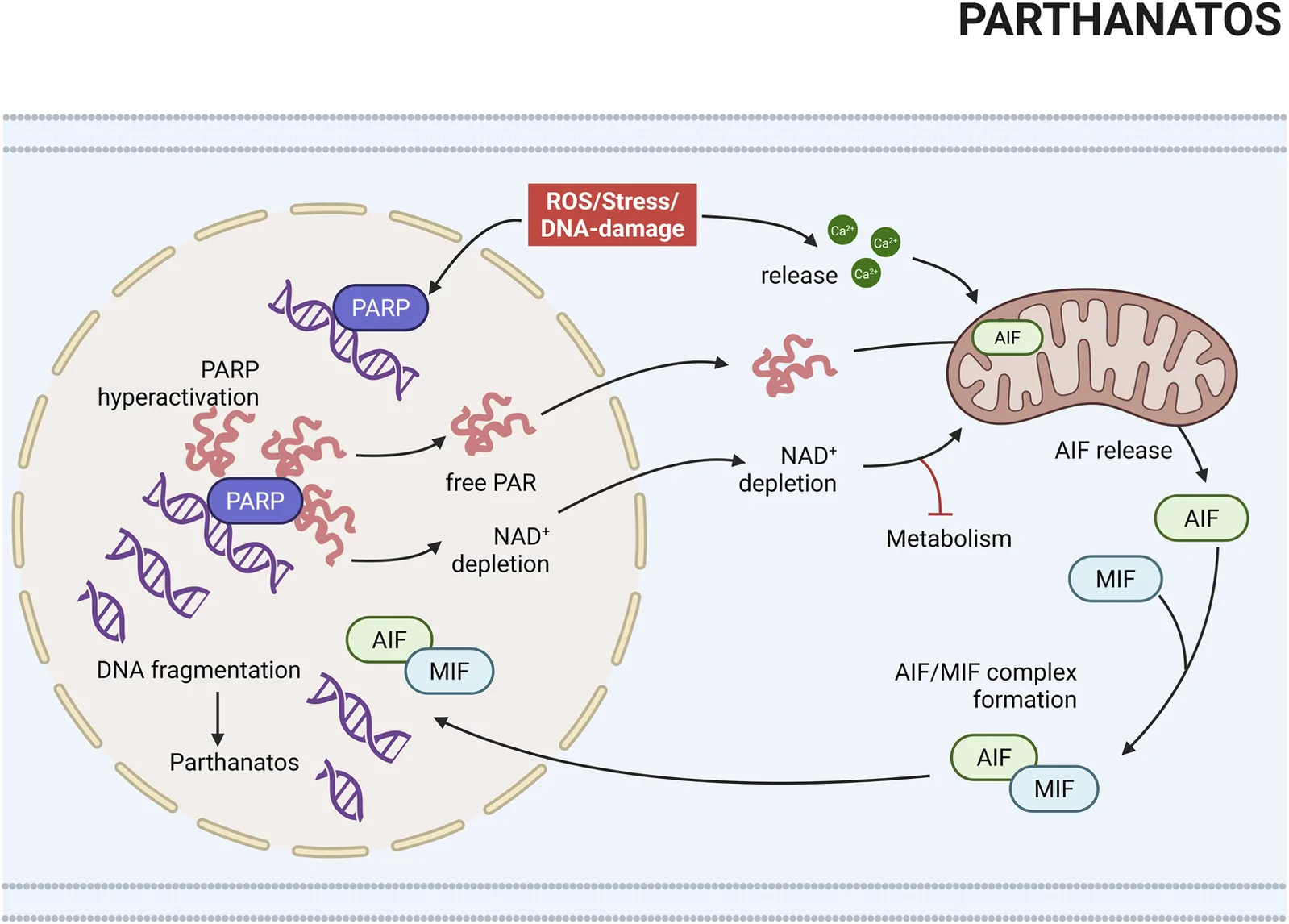
Parthanatos in OC. Oxidative stress or DNA damage hyperactivates PARP, depleting NAD^+^ and triggering AIF/MIF release from mitochondria. Nuclear translocation of AIF/MIF drives DNA fragmentation and cell death. Abbreviations: PAR, poly-ADP-ribose; NAD^+^, nicotinamide adenine dinucleotide.

Parthanatos is a form of RCD initiated by hyperactivation of PARP-1, with molecular mechanisms and morphological characteristics distinct from other cell death pathways (Figure 7) (Wang et al., 2016). Under severe DNA damage, PARP-1 becomes aberrantly activated and catalyzes extensive synthesis of PAR polymers. The generated PAR chains bind to apoptosis-inducing factor (AIF), triggering its release from mitochondria and subsequent nuclear translocation. Within the nucleus, AIF interacts with macrophage migration inhibitory factor (MIF) to form the AIF/MIF complex, which exhibits DNA-degrading activity and induces large-scale DNA fragmentation, ultimately leading to cell death (Wang et al., 2016). Morphologically, parthanatos is characterized by nuclear condensation, extensive DNA fragmentation, and loss of plasma membrane integrity, without significant cell swelling or apoptotic body formation (Yu et al., 2002). Given the critical roles of PARP-1 and associated molecules in parthanatos, they represent potential targets for therapeutic intervention in cancer and other diseases.

Regarding OC, recent studies have constructed a prognostic risk model based on parthanatos-related genes through multi-omics analysis, demonstrating its close association with the tumor immune microenvironment. Notably, the key gene transforming growth factor-beta-induced protein was validated *in vitro* to promote OC cell proliferation and invasion, suggesting its potential as a therapeutic target (Qi et al., 2025). Another study revealed that cisplatin can induce tumor cell death through activation of the PARP-1-mediated parthanatos pathway. Intriguingly, the PARPi PJ34, while blocking this pathway to protect auditory cells from cisplatin-induced damage, conversely enhances cisplatin chemosensitivity in OC cells, uncovering the dual role of this pathway in tissue-specific regulation (Nong et al., 2025). Although research on parthanatos in OC remains at a preliminary stage, its distinctive mode of cell death exhibits significant potential in DNA damage repair and immune modulation, foreshadowing broad prospects for therapeutic applications in OC management.

### 4.2 Disulfidptosis

**FIGURE 8:**
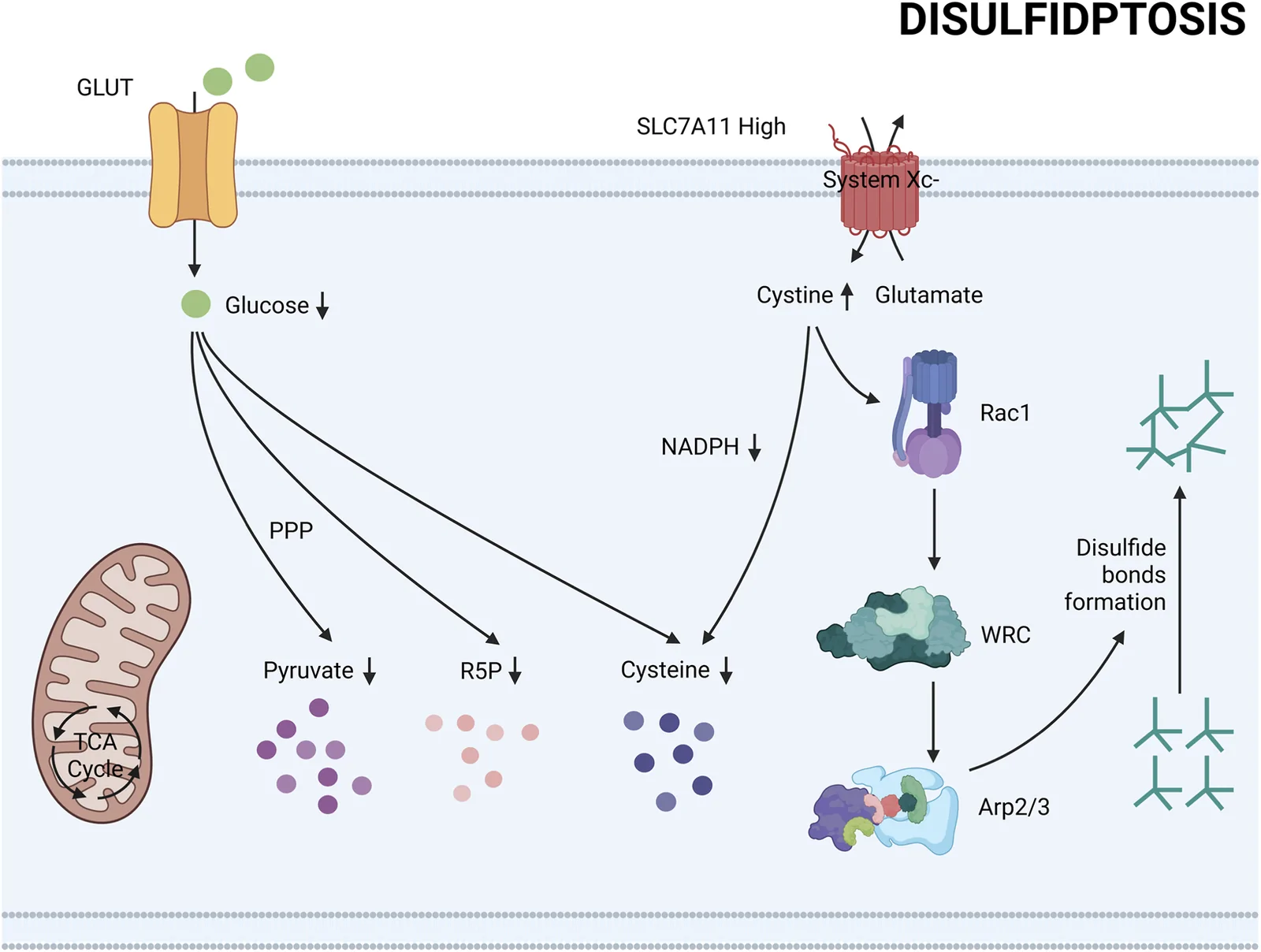
Disulfidptosis in OC. In SLC7A11-high cells, glucose deprivation depletes NADPH and impairs cystine reduction. Cumulative disulfide stress collapses the actin cytoskeleton. Abbreviations: SLC7A11, solute carrier family 7 member 11; PPP, pentose phosphate pathway; R5P, ribose-5-phosphate; TCA, tricarboxylic acid; WRC, WAVE regulatory complex; Arp2/3, actin-related protein 2/3 complex; Rac1, Ras-related C3 botulinum toxin substrate 1.

As previously described, SLC7A11, functioning as a cystine transporter, promotes extracellular cystine uptake to support glutathione synthesis and enhance GPX4-mediated antioxidant defense, thereby suppressing ferroptosis. However, recent studies have revealed that under glucose starvation conditions, SLC7A11 overexpression paradoxically induces a novel form of programmed cell death termed disulfidptosis (Figure 8) (Liu X. et al., 2023). The distinctive feature of disulfidptosis lies not merely in NADPH depletion, but in concomitant intracellular disulfide bond stress and collapse of the actin cytoskeleton (Mao et al., 2024) At the molecular level, glucose starvation combined with high SLC7A11 expression leads to NADPH exhaustion, preventing cystine reduction and resulting in aberrant intracellular disulfide bond accumulation and actin filament cross-linking disruption, ultimately culminating in cell death (Mao et al., 2024). This discovery provides a novel therapeutic strategy, as cancer cells frequently upregulate SLC7A11 to cope with oxidative stress, rendering them vulnerable to disulfidptosis under glucose deprivation (Qiu et al., 2025).

Scoring systems based on disulfidptosis-related genes have demonstrated significant potential in prognostic prediction for OC patients, as well as in optimizing selection of immunotherapy and chemotherapy regimens, providing important molecular biological evidence for clinical decision-making (Liu S. et al., 2024). Further investigations have revealed that depleting intracellular NADPH with 6-aminonicotinamide (6-AN) leads to aberrant cysteine accumulation, thereby inducing abnormal disulfide bond cross-linking of the actin cytoskeleton in SLC7A11-high OC cells, ultimately triggering disulfidptosis in a specific manner (Fan et al., 2025). This discovery opens new avenues for overcoming current therapeutic challenges.

## 5 Nanotechnology-based delivery platforms for RCD modulation in OC

OC exhibits one of the highest mortality rates among gynecological malignancies, primarily attributable to late-stage diagnosis, extensive peritoneal dissemination, and multidrug resistance (MDR) (Xu et al., 2026). To circumvent the limitations of conventional apoptosis-targeted therapeutics, nanotechnology has evolved from passive drug carriers into intelligent theranostic systems capable of precisely modulating non-apoptotic RCD. The core advantages of such nano-platforms include: (i) enhanced drug accumulation at peritoneal micrometastatic lesions through active ligand-mediated targeting (Xu et al., 2026); (ii) effective reversal of MDR by evading P-glycoprotein efflux pumps or co-delivering resistance-associated siRNAs (El-Mallul et al., 2025); and (iii) integration of multimodal imaging probes to enable intraoperative navigation and real-time therapeutic monitoring (Liu A. et al., 2025; Tian et al., 2026). Accordingly, contemporary research has shifted beyond the mere delivery of conventional chemotherapeutics toward engineering nanoparticles that actively induce diverse non-apoptotic RCD modalities, with the aim of overcoming apoptosis-resistance bottlenecks (Luo et al., 2025).

### 5.1 Lipid and polymer carriers: targeted delivery and autophagy modulation

Liposomes and polymeric nanoparticles exhibit excellent biocompatibility and versatile encapsulation capacity for both hydrophilic and hydrophobic therapeutics, serving as foundational platforms for nanotherapeutic interventions in OC (El-Mallul et al., 2025). These nanocarriers ameliorate the tissue distribution and pharmacokinetic profiles of poorly soluble drugs, enabling tumor-targeted delivery and sustained release (Xu et al., 2026). Extracellular vesicles, as naturally derived nanocarriers, possess inherently low immunogenicity and intrinsic targeting capabilities, rendering them well-suited for the delivery of chemotherapeutic agents or siRNAs with enhanced targeting efficiency and attenuated off-target toxicities (Yun et al., 2025). In the context of autophagy modulation, aptamer-functionalized nanoparticles offer distinct advantages. Notably, nucleic acid aptamer-conjugated zinc ferrite nanoparticles have been demonstrated to enhance cisplatin sensitivity in OC cells through the induction of cytotoxic autophagy, thereby effectively reversing chemoresistance (Zhu et al., 2024). This paradigm shifts autophagy from a pro-survival to a pro-death modality, offering a promising strategy to circumvent drug resistance.

### 5.2 Inorganic and hybrid nanomaterials: ferroptosis induction and immune activation

Inorganic nanoparticles incorporating iron or other transition metals have emerged as potent tools for inducing ferroptosis. Ultrasound-responsive Bi_2_MoO_6_-MXene heterojunctions efficiently generate ROS upon sonodynamic activation, triggering ferroptosis in OC cells and eliciting immunogenic cell death (ICD), thereby achieving synergistic effects between direct tumoricidal action and immune stimulation (Cheng et al., 2024). Multifunctional biomimetic nanosystems integrating magnetic targeting and glucose oxidase-mediated starvation therapy have demonstrated superior theranostic potential in preclinical models through precisely orchestrated ferroptosis induction (Pan et al., 2025). Furthermore, metal-organic frameworks, leveraging their high porosity and metal node-catalyzed ROS generation, have been extensively exploited to augment ferroptosis-mediated antitumor therapy by dismantling the antioxidant defense systems of cancer cells (Ma et al., 2026).

### 5.3 Copper-based nanomedicines: multimodal RCD synergy

Copper-based nanomedicines exploit the elevated copper demand of OC cells to achieve tumor-responsive copper ion release via GSH-triggered mechanisms, thereby cooperatively inducing multiple RCD modalities including apoptosis, ferroptosis, and cuproptosis (Ma L. et al., 2025). The underlying mechanism involves copper ion-elicited mitochondrial dysfunction and lipid peroxidation, which act synergistically to generate overwhelming ROS storms that surpass cellular repair capacity (Luo et al., 2026). This multimodal death-induction strategy effectively circumvents compensatory activation of alternative survival pathways upon single-pathway inhibition, rendering it particularly suitable for highly heterogeneous and drug-resistant OC.

### 5.4 Intelligent responsive nanoplatforms: precision drug release and immune reprogramming

Next-generation stimulus-responsive nanoplatforms—such as DNA nanosponge systems and ultrasound-driven nanozymes—are selectively activated under specific tumor microenvironment (TME) conditions (e.g., acidic pH, elevated ROS), enabling the induction of multimodal RCD to break the “whack-a-mole” paradigm of drug resistance in tumor recurrence (Kejun et al., 2025; Luo et al., 2026)). Gold nanocarriers co-delivering doxorubicin and erbB2-siRNA simultaneously suppress resistance pathways and transport cytotoxic payloads, further synergizing with immune checkpoint inhibitors to convert the immunologically “cold” OC microenvironment into an inflamed “hot” state (Kotcherlakota et al., 2017). Moreover, nanoparticles enhance immunotherapy responses by inducing ICD to release damage-associated molecular patterns, thereby augmenting antigen presentation and recruiting immune cells (Li Y. et al., 2024; Hu J. et al., 2025). Collectively, these advances demonstrate that nanotechnology is emerging as a pivotal strategy for overcoming chemoresistance and potentiating immunotherapeutic efficacy in OC.

In summary, lipid/polymeric carriers, inorganic materials, and copper-based nanomedicines modulate RCD through distinct yet complementary mechanisms—autophagy reprogramming, ferroptosis induction, and multimodal synergy, respectively—while intelligent responsive strategies endow these systems with TME-activated precision. Although challenges persist regarding stability, targeting efficiency, and biosafety, their integration with immunotherapy has demonstrated substantial potential in overcoming chemoresistance and reshaping the immune microenvironment. Future efforts should prioritize mechanism-convergent engineering and clinical translation.

## 6 Predictive biomarkers for RCD-targeted therapies in OC

As previously discussed, nanotechnology platforms have provided a versatile arsenal for precision modulation of RCD. However, the marked intertumoral and intratumoral heterogeneity of OC poses a critical challenge in clinical translation: how to accurately identify patient subpopulations most likely to benefit from RCD-targeted therapies. Reliable predictive biomarkers constitute the prerequisite for precision patient stratification. Based on existing evidence, relevant biomarkers can be categorized into clinically validated and clinically promising classes.

Clinically validated biomarkers primarily include BRCA1/2 mutation status, homologous recombination deficiency (HRD) scores, and folate receptor α (FRα). BRCA1/2 mutations directly abrogate homologous recombination repair capacity, serving as robust predictive determinants of PARPi efficacy (DiSilvestro et al., 2023). HRD scores, by quantifying genomic instability, enable the identification of a broader spectrum of homologous recombination repair-deficient patients (Khokhlova et al., 2025). Collectively, these markers reflect impaired DNA double-strand break repair capacity, conferring exquisite sensitivity to PARPi. Multiple phase III trials have demonstrated that patients stratified by these biomarkers derive significant progression-free survival benefit from PARPi maintenance therapy (Yi et al., 2022; DiSilvestro et al., 2023). Additionally, FRα overexpression in a subset of high-grade serous OC has established its utility as a patient selection biomarker for FRα-targeted antibody–drug conjugates, with phase III trials confirming survival benefits in FRα-positive platinum-resistant patients (Moore et al., 2023; Coleman et al., 2024). Among clinically promising biomarkers, ferroptosis-related molecules GPX4 and ACSL4 have been implicated in OC prognosis and chemosensitivity, representing the most intensively investigated candidates (Taki et al., 2026). Regarding cuproptosis, FDX1 expression levels may predict tumor cell sensitivity to copper ionophores; however, current evidence derives predominantly from cell line studies, and its clinical value in OC awaits systematic validation (Zhang et al., 2024). In summary, FDA-approved biomarkers capable of directly guiding RCD-targeted therapeutic decisions in OC remain scarce, underscoring the imperative for rigorous clinical investigation of these candidate molecules.

## 7 Summary of OC clinical trials targeting RCD modalities

Although activators and inhibitors of various RCD subtypes have been extensively reported in recent years, clinical trials in OC remain predominantly focused on apoptosis modulation (Table 1), with clinical evidence for other RCD modalities still insufficient. As mechanistic understanding continues to advance, more clinical trials are anticipated to systematically evaluate the therapeutic potential of diverse RCD modalities in OC.

**TABLE 1:** OC clinical trials targeting RCD modalities.

Nct number	Drug	Function	Phase	First posted
NCT00756847	XL147 + Paclitaxel + Carboplatin	Induce apoptosis	Phase 1	2008-09-22
NCT01989546	BMN 673	Induce apoptosis	Phase 2	2013-11-21
NCT02312661	Metformin + Carboplatin/Paclitaxel	Induce apoptosis	Phase 1	2014-12-09
NCT03042702	Kevetrin	Induce apoptosis	Phase 2	2017-02-03
NCT05358639	Olaparib + Navitoclax	Induce apoptosis	Phase 1	2022-05-03
NCT06048367	CNSI-Fe(II)	Induce ferroptosis	Phase 1	2023-09-21

Sourced from https://clinicaltrials.gov/, July 2026.

## 8 Conclusions and perspectives

OC represents the leading cause of mortality among gynecological malignancies. Its pronounced inter- and intra-tumoral heterogeneity, coupled with the intricate TME, poses substantial challenges to conventional therapeutic strategies and contributes to unfavorable patient outcomes. Rapid advances in the study of RCD have revealed that dysregulated RCD serves as a critical driver in the initiation and progression of OC, offering a wealth of potential molecular targets for therapeutic intervention. This review systematically delineates the mechanistic roles of eight RCD subtypes in OC, ranging from classical apoptosis to emerging modalities such as disulfidptosis, and comprehensively summarizes research progress on small-molecule compounds targeting these pathways (Table 2), with the aim of establishing an integrated therapeutic framework centered on RCD modulation. Our core perspectives are threefold: First, individual RCD subtypes do not operate in isolation but constitute a highly interconnected regulatory network through shared modulatory molecules and signaling cascades. Second, RCD-targeted therapeutic strategies are undergoing a paradigm shift from “single-pathway induction” toward “coordinated multi-pathway modulation,” which has emerged as the central strategy to overcome chemoresistance and achieve synergistic antitumor efficacy. Third, RCD-related research is progressively transitioning from phenomenological description toward precise therapeutic regulation and clinical translation, driven by the convergent development of small-molecule compounds, natural products, and novel nano-delivery systems, alongside the discovery and application of predictive biomarkers. Nevertheless, the central challenge in the field has shifted from elucidating “what RCD is” to addressing “how to optimally exploit RCD for therapeutic purposes”—a transition that imposes more stringent demands on drug performance and exposes persistent barriers in the translation from basic research to clinical application.

**TABLE 2:** Therapeutic targets connected with RCD in OC.

RCD	Target	Compound	Model (*in vitro*; *in vivo*)	Mechanism	References
Apoptosis	Bcl-2 family proteins	IS21, S1, ABT-737	OVCAR 3/5/8/420/432/433, SKOV3; Subcutaneous SKOV3 xenograft in BALB/c nude mice, *Ex vivo* HGSOC patient tumor explants treated	BH3 mimetic	Lheureux et al. (2015), Xiang et al. (2016), Valentini et al. (2023)
		Emd-D, Dendritic curcumin, Isoaaptamine	OVHM, SKOV3, OVCAR 3, A2780/cis, PA-1; Subcutaneous OVHM xenograft in BALB/c mice	Upregulates Bax/Bcl-2 expression	Su et al. (2025a), Zarei et al. (2025), Zhao et al. (2025b)
	IAPs	LCL161, AT406, Birinapant	UWB-1289, UWB1289-RES, ES2, A2780, A2780/cis, OVCAR 3, CAOV3, OV90, 3D Organoid Bioassay, 2D Monolayer Cell Culture; NSG mouse with HGSOC PDX (subcutaneous), NCR nude mouse with intraperitoneal ES2 xenograft, PDX model in NSG mice (subcutaneous implantation of platinum-resistant EOC2 tumor cells)	Smac mimetic	Rada et al. (2018), Cremona et al. (2022), Singh et al. (2022)
	DRs	ONC201, Quinacrine, Quercetin	OVCAR 3/4/5/8, IGROV-1, SKOV3, A2780, TOV-21G; KpB transgenic mouse (p53/Brca1-deficient), A2780-luc intraperitoneal xenograft model in NSG mice, Athymic nude mice bearing subcutaneous SKOV3 xenografts	Upregulates DR5 expression	Allen et al. (2013), Yi et al. (2014), Liang et al. (2020), Fan et al. (2022)
	JNK/p38 MAPK	SB203580	HEY, A2780; Subcutaneous xenograft model in NCG mice	Inhibits p38	Sun et al. (2023)
		Piperine, Sideroxylin	A2780, OSE, ES2, OV90	Activates JNK/p38 MAPK	Park et al. (2018), Si et al. (2018)
		Tanshinone IIA	TOV-21G, SKOV3, OVCAR 3	Activates p38 MAPK and downregulates survivin expression	Lin et al. (2015)
	PI3K/AKT/mTOR	Methyl lucidone	OVCAR 8, SKOV3	Inhibits PI3K/Akt/mTOR	Yoon et al. (2020)
		Diosgenin	OVCAR 3, SKOV3	Upregulates PTEN expression and inhibits PI3K/Akt/mTOR	Fang et al. (2024)
		Isojacareubin	HEY, ES-2	Inhibits PI3K/Akt/mTOR and MAPK/ERK	Tang et al. (2020)
	p38/p53	Triptonide	A2780, SKOV3	Activates p38/p53	Lou et al. (2024)
	Ca^2+^/CAMKK2/Akt/mTOR	Sodium citrate	A2780, SKOV3; Subcutaneous SKOV3 xenograft in BALB/c nude mice	Inhibits Ca^2+^/CAMKK2/Akt/mTOR	Wu et al. (2024)
	SCD1	Agrimonolide	A2780, SKOV3; Subcutaneous SKOV3 xenograft in BALB/c nude mice	Inhibits SCD1 expression	Liu et al. (2022d)
Autophagy	PI3K/AKT/mTOR	WX390, MK8722, MHY2245	ES2, OVISE,A2780, OV90, SKOV3, OVCAR 3; Subcutaneous PDX model in NCG mice, Subcutaneous A2780 or OV90 xenograft in BALB/c nude mice, Subcutaneous SKOV3 xenograft in BALB/c nude mice	Inhibits PI3K/AKT/mTOR	Tae et al. (2020), Wang et al. (2024a), Zheng et al. (2025a)
		Icariin	SKVCR	Activates AKT/mTOR/ATG5	Jiang et al. (2019)
		Paeonol, Daphnetin	A2780, SKOV3, OVCAR8; Subcutaneous A2780 xenograft in BALB/c nude mice	Inhibits AKT/mTOR	Gao et al. (2019)
​	ULK1	MRT68921	OVCAR3, OVCAR4, OVCAR8, COV318, COV362, CaOV3, iOvCa147, iOvCa256, iOvCa360	Inhibits ULK1	Singha et al. (2020)
		Crizotinib + Olaparib	OVCAR8, A2780, OC316, SKOV3, HEY, OVCAR 3, IGROV1OR, OC316OR; OVCAR8 intraperitoneal xenograft in athymic nude mice	Inhibits AKT/mTOR/ULK1	Santiago-O'Farrill et al. (2024)
		Cinnamaldehyde	SKOV3, HEY; HEY subcutaneous xenograft in BALB/c nude mice	Activates AMPK/ULK1/Beclin1	Yi et al. (2026)
	ATG proteins	Antrodia salmonea	SKOV3, A2780	Inhibits ATG4B	Yang et al. (2019)
	Lysosomes	Elaiophylin, Dihydrotanshinone I, Costunolide	SKOV3, OVCAR3, A2780, CaOV3, SW626, ES2, HO8910PM, HEY; SKOV3 subcutaneous xenograft in BALB/c athymic nude mice, ES2 subcutaneous xenograft in BALB/c athymic nude mice	Disrupts lysosomal function	Zhao et al. (2015), Liang et al. (2023), Sun et al. (2025)
	FAK	E2	PA-1, ES-2, OVCAR 3; PA-1 subcutaneous xenograft in BALB/c athymic nude mice	Inhibits FAK	Feng et al. (2024)
	lncRNA LINC00123/p65	Berberine	SKOV3, HEY; HEY subcutaneous xenograft in BALB/c athymic nude mice	Interferes with the interaction between lncRNA LINC00123 and p65	Yan et al. (2024)
	Mortalin	SHetA2	A2780, SKOV3, OV90, OVCAR3, CAOV3; OV90 subcutaneous xenograft in SCID mice	Activates PINK1/Parkin	Chandra et al. (2025)
Necroptosis	RIPK1/RIPK3/MLKL	PTC-209, Sulfasalazine, Fisetin, GA	CP20, OVCAR 4, SKOV3, SKOV3/DDP, A2780, OVCAR 3; A2780 subcutaneous xenograft in BALB/c nude mice	Activates RIPK1-RIPK3-MLKL	Dey et al. (2016), Liu et al. (2022c), Liu et al. (2024a), Li et al. (2025)
Pyroptosis	GSDM	BI 2536	A2780; A2780 subcutaneous xenograft in BALB/c nude mice	Activates caspase-3/GSDME	Huo et al. (2022)
		Nobiletin	A2780, OVCAR 3	Activates GSDMD/GSDME	Zhang et al. (2020)
		α-NETA	Ho8910, Ho8910PM, A2780, SkOV3, HEY; SKOV3 subcutaneous xenograft in BALB/c nude mice	Activates GSDMD/caspase-4	Qiao et al. (2019)
	Inflammasomes	Statins	RMG1, OVCA429, ES2, OVISE, OVTOKO, SKOV3, TOV21; Orthotopic OCCC xenograft in NSG mice, Arid1a^−/−^; Pik3ca^H1047R^ transgenic OCCC mouse model, Humanized BLT mouse model bearing ARID1A-mutant OCCC PDX	Activates NLRP3/caspase-1/GSDMD	Zhou et al. (2023)
		Azacytidine + Talazoparib	TYK-nu, OVCAR 4, A2780, CP70, HeyC2, C272, OVCAR 8, OV2008, HEYA2; TYK-nu subcutaneous xenograft in NSG mice	Activates STING	Stojanovic et al. (2025)
Ferroptosis	GPX4	ML210 + Olaparib, Shikonin + Cisplatin, Isoginkgetin	UWB1.289, A2780, SKOV3, OVCAR 3, HCC1937, OVCAR 4; A2780-BRCA1 KO subcutaneous xenograft in BALB/c nude mice, A2780/DDP subcutaneous xenograft in BALB/c nude mice	Downregulates GPX4 expression	Ni et al. (2023), Xie et al. (2024), Hu et al. (2025a)
​	DHODH	Eribulin	RMG-1, ES-2; RMG-1 subcutaneous xenograft in BALB/c nude mice	Downregulates DHODH expression	Azumi et al. (2024)
	SLC7A11	Icariside II, Lidocaine, Artesunate	SKOV3, OVCAR 3; SKOV3 subcutaneous xenograft in BALB/c nude mice	Downregulates SLC7A11 expression	Sun et al. (2021), Nie et al. (2025), Yuan et al. (2025)
	GSH	TF3	A2780/CP70, OVCAR3	Decreases GSH levels	Pan et al. (2018)
		Daphnetin	A2780, SKOV3; A2780 subcutaneous xenograft in BALB/c nude mice	Inhibits NQO1 and decreases GSH/GSSG ratio	Ma et al. (2024)
		Losartan + RSL3	OCAR8, A2780, COC1; OCAR8 subcutaneous xenograft in athymic nu/nu nude mice	Inhibits PAX8-GCLC and downregulates GSH synthesis	Luo et al. (2024)
	SPHK1/NF-κB/NRF2	PF-543	SKOV3, OVCAR8; OVCAR8 subcutaneous xenograft in BALB/c athymic nude mice	Inhibiting SPHK1 restricts NF-κB-activated NRF2 transcription	Teng et al. (2025)
	Sirtuin 1	NTX-301	OVCAR5, OVCAR4, SKOV3, COV362; OVCAR5 subcutaneous xenograft in athymic nude mice, HGSOC patient-derived xenograft in NSG mice	Upregulates sirtuin 1 transcription	Wang et al. (2024b)
	GLUT1	LA-GlcN	SKOV3; SKOV3 subcutaneous xenograft in BALB/c nude mice	Targets GLUT1	Chen et al. (2022)
Cuproptosis	FDX1	Elesclomol	SKOV 3, A2780; SKOV-3 subcutaneous xenograft in BALB/c nude mice	Upregulates FDX1 expression	Zou et al. (2025)
		Disulfiram	SKOV 3, A2780; SKOV-3 subcutaneous xenograft in NOD/SCID mice	Downregulates FDX1 transcription upon binding to copper	Gan et al. (2023)
		Anisomycin	HuOCSCs; HuOCSCs subcutaneous xenograft in immunodeficient mice	Downregulates FDX1 transcription	Nie et al. (2022)
Parthanatos	PARP	Cisplatin	HEY, TOV112D	Activates PARP-1	Nong et al. (2025)
Disulfidptosis	NADPH	6-AN	OVCAR3, TOV-21G, A2780; OVCAR3 subcutaneous xenograft in BALB/c nude mice	Depletes NADPH	Fan et al. (2025)

Drugs serve as the principal conduit bridging mechanistic understanding and clinical efficacy. The inherent limitations of current RCD modulators in terms of druggability directly epitomize this translational impediment. Synthetic small molecules offer well-defined targets and amenable structural optimization, yet frequently encounter challenges including insufficient selectivity, off-target toxicity, and acquired resistance (Islam et al., 2023). Natural products, by contrast, possess distinctive advantages in orchestrating complex RCD networks through their polypharmacological properties and relatively favorable toxicity profiles; however, their clinical advancement is severely constrained by poor aqueous solubility, low bioavailability, rapid *in vivo* metabolism, the mechanistic complexity arising from multi-target engagements, and difficulties in quality control standardization (Muttiah and Abdullah, 2025). Notably, the limitations of these two drug categories exhibit complementary characteristics—the target precision of synthetic compounds and the multi-target synergistic effects of natural products can be integrated through rational combination regimens or structure optimization based on natural product scaffolds, thereby potentiating therapeutic efficacy while mitigating toxicity (Nazam et al., 2023). Nevertheless, regardless of drug classification, the translational bottleneck has extended beyond the intrinsic attributes of the agents themselves to encompass a critical knowledge deficit regarding optimal patient selection, therapeutic timing, and the sequential orchestration of combination protocols.

These knowledge deficits manifest as three interrelated limitations. First, at the model level, the vast majority of RCD modulation strategies have been validated primarily in OC cell lines and subcutaneous xenograft models, which inadequately recapitulate the spatiotemporal heterogeneity of human OC and its distinctive peritoneal metastatic microenvironment—an inadequacy that contributes to a disproportionately high rate of clinical translation failure. Second, at the mechanistic level, a substantial body of research remains confined to phenotypic characterization, lacking systematic investigation into the precise targets, off-target effects, and resistance mechanisms of RCD modulators within specific OC molecular subtypes. Third, at the clinical level, clinically validated predictive biomarkers remain to be established, impeding the implementation of precision patient stratification—it remains unclear which molecular subtypes are most likely to derive benefit from specific RCD-targeted therapies, and the optimal sequencing and combinatorial strategies for such interventions have yet to be defined. These three limitations are mutually reinforcing and collectively constitute a fundamental barrier to clinical translation.

The resolution of these obstacles delineates the future priorities for this field. First, leveraging single-cell sequencing, spatial transcriptomics, and related technologies to dynamically map the temporal sequence and functional coupling of distinct RCD subtypes within primary OC lesions and their peritoneal metastatic microenvironment, thereby elucidating the molecular switches governing death pathway transitions, is essential to establish a rational foundation for the design of precisely timed combination therapeutic regimens (Hu J. et al., 2025). Second, concerted efforts should be directed toward identifying nodal regulatory factors within the RCD network that may serve as “pan-RCD modulators,” integrating structure-based drug design, PROTACs, molecular glues, and other emerging discovery modalities, while harnessing biomimetic nano-delivery systems to achieve targeted delivery and controlled release of RCD modulators (Isert et al., 2023; Eladl, 2025; Lin J. et al., 2025). Third, the establishment of robust biomarker panels is imperative to enable multi-omics-guided patient stratification and to explore reversal strategies for acquired resistance to RCD-targeted therapies. Furthermore, given that the distinctive peritoneal metastatic microenvironment of OC may influence RCD sensitivity (Lane et al., 2010), future drug screening and mechanistic investigations should be conducted in more physiologically relevant three-dimensional organoid or microfluidic chip models to enhance the predictive validity of preclinical studies. Ultimately, the integration of multi-omics, chemical biology, intelligent nanomedicine, and precision medicine concepts holds promise for translating RCD regulatory mechanisms into innovative combination therapeutic strategies, thereby delivering tangible clinical benefits to patients with OC.
